# The a subunit isoforms of V-ATPase are involved in glucose-dependent trafficking of insulin granules

**DOI:** 10.1038/s41598-025-02997-7

**Published:** 2025-05-30

**Authors:** Mizuki Sekiya, Mayumi Nakanishi-Matsui, Naomi Matsumoto, Iwao Takahashi, Tomohito Hanasaka, Eri Ishiyama-Matsuura, Makoto Araki, Tomohiko Wakayama, Koji Nata

**Affiliations:** 1 Division of Biochemistry, School of Pharmacy, Iwate Medical University, Idaidori 1-1-1, Shiwa, Yahaba, Iwate 028-3694 Japan; 2 Center for Basic Medical Research, International University of Health and Welfare, Narita, Chiba 286-8686 Japan; 3 Division of Molecular and Cellular Pharmacology, School of Pharmacy, Iwate Medical University, Yahaba, Iwate 028-3694 Japan; 4 Technical Support Center for Life Science Research, Iwate Medical University, Yahaba, Iwate 028-3694 Japan; 5 Department of Biochemistry, Meiji Pharmaceutical University, Noshio, Kiyose, Tokyo 204-8488 Japan; 6 Department of Histology, Graduate School of Medical Science, Kumamoto University, Kumamoto, 860-8556 Japan; 7 Division of Medical Biochemistry, School of Pharmacy, Iwate Medical University, Yahaba, Iwate 028-3694 Japan

**Keywords:** V-ATPase, a subunit isoforms, Insulin secretion, Membrane trafficking, Rab27A, Biochemistry, Membrane trafficking

## Abstract

In pancreatic β cells, insulin granules move toward the plasma membrane to secrete insulin upon glucose stimulation, but the amount of secreted insulin is only a small portion of the total, and many granules do not release insulin. Here, using MIN6 cells derived from mouse pancreatic β cells, we observed that granules that moved toward the plasma membrane returned to the inner area after the stimulation was removed. This back-and-forth trafficking is likely important for strict regulation of insulin secretion in response to the blood glucose level. However, the mechanism was largely unknown. We found that “back” (inward) and “forth” (outward) trafficking was reduced in cells with knockdown of the a2 and a3 subunit isoforms of the proton pump V-ATPase, respectively. Interestingly, the amount of secreted insulin was increased in a2 knockdown cells. Both a2 and a3 interacted with GDP-bound form Rab27A, a member of the Rab small GTPase family that regulates insulin secretion. These results indicate that a2 and a3 are involved in back-and-forth trafficking of insulin granules, respectively. The a subunit isoforms of V-ATPase seem to determine the direction of insulin granule trafficking dependent on the glucose level.

## Introduction

Insulin secretion from pancreatic β cells is essential to regulate the blood glucose level. Insulin secretion is biphasic; there is an early phase within a few minutes after glucose stimulation and a subsequent late phase for sustained lower-level secretion. It is known that resident-type insulin granules located beneath the plasma membrane with or without glucose stimulation are responsible for early phase secretion^1–4^. Recent studies revealed that granules located in the inner region that move toward and fuse with the plasma membrane upon glucose stimulation are also responsible for early phase secretion^2^. We are interested in glucose-dependent trafficking of insulin granules.

Although insulin granules move close to the plasma membrane when the glucose concentration is high, the portion of released insulin is small relative to the total amount: the level of released insulin is less than 10% in human and mouse and never more than 15% in a mouse pancreatic β cell line, MIN6^5–11^. These observations suggest that many insulin granules move to cell periphery but do not release insulin, so-called insulin-unreleased granules. What is the fate of these granules? Using MIN6 cells, we found that insulin-unreleased granules returned to the cell interior within 5 min after the stimulation was removed. This rapid return of insulin-unreleased granules seems important to prevent unnecessary insulin release at the plasma membrane and thus to promptly turn off insulin release when the blood glucose concentration decreases.

Recent studies of insulin secretion revealed that Rab27A, a member of the Rab small GTPase family involved in various secretion processes, controls insulin secretion^12,13^. Rab27A on insulin granules recruits a specific set of effectors, such as granuphilin, exophilin-7, and exophilin-8, which enables multiple pathways of insulin exocytosis^14–17^. After membrane fusion between insulin granules and the plasma membrane, the membrane of insulin granules is recovered from the plasma membrane through glucose-induced endocytosis^18^. GTP-bound Rab27A is converted to the GDP-bound form at the plasma membrane by EPI64 and IQGAP1, and coronin3, the specific effector of GDP-bound Rab27A, induces endocytosis^19–21^. This glucose-induced endocytosis seems important to recycle the membrane of insulin granules and to maintain the cell volume. However, the rapid return of insulin-unreleased granules that we report in this study is completely different from high glucose-induced endocytosis of insulin granule membranes, and the molecular mechanism underlying this rapid return was largely unknown.

**Fig. 1:**
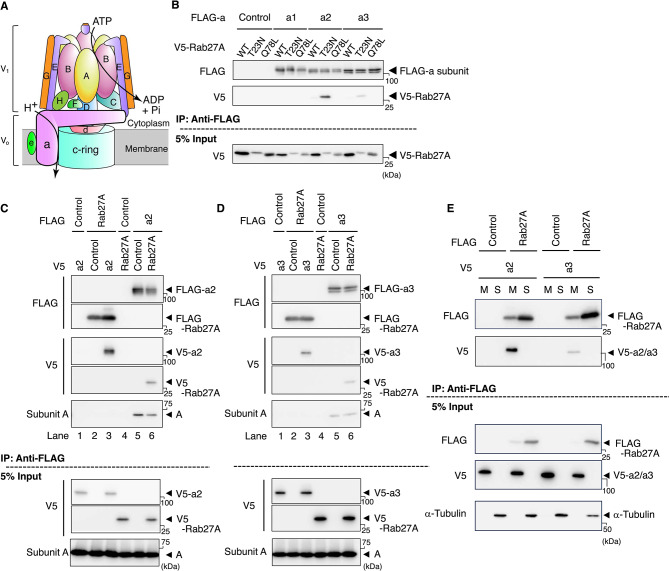
Interaction of a subunit isoforms with Rab27A and A subunit. (**A**) Schematic illustration of the subunits of the mammalian V-ATPase. (**B**) Interaction of a subunit isoforms with Rab27A. FLAG-tagged a isoforms and various V5-fused forms of Rab27A were co-expressed in HEK293T cells. The cells were lysed, and lysates were immunoprecipitated with an anti-FLAG antibody. The precipitates were analyzed using antibodies against FLAG (upper panel) and V5 (middle panel). As a control, cells were co-transfected with an empty vector and a recombinant plasmid harboring V5-fused Rab27A (Control). WT, T23N, and Q78L indicate wild-type, GDP-bound form, and GTP-bound form Rab27A, respectively. In addition, 5% of the cell lysate was subjected to western blotting with an anti-V5 antibody (lower panel). Original blots are presented in Supplementary Fig. 7. The blots in (**B**) were cut horizontally at ~ 60 kDa. Upper and lower membranes were subjected to hybridization with anti-FLAG and anti-V5 antibodies, respectively. A full-length membrane was cut because the amount of protein in immunoprecipitation samples was insufficient to prepare multiple full-length membranes. (**C**) Interaction of the a2 subunit and Rab27A with the A subunit in V_1_. FLAG-Rab27A(T23N) (lanes 1–3) and FLAG-a2 (lanes 4–6) were co-expressed with V5-a2 and V5-Rab27A(T23N), respectively. Immunoprecipitation was performed as described in (**B**). Original blots are presented in Supplementary Fig. 8. **D** Interaction of the a3 subunit and Rab27A with the A subunit in V_1_. FLAG-Rab27A(T23N) (lanes 1–3) and FLAG-a3 (lanes 4–6) were co-expressed with V5-a3 and V5-Rab27A(T23N), respectively. Immunoprecipitation was performed as described in (**B**). Original blots are presented in Supplementary Fig. 8. (**E**) Presence of a2 and a3 isoforms and Rab27A in the membrane fraction. FLAG-Rab27A(T23N) and V5-a isoforms were co-expressed in HEK293T cells. The cells were lysed and centrifuged at 100,000 × g for 30 min to separate the membrane (M) and soluble (S) fractions. Immunoprecipitation was performed as described in (**B**). α-Tubulin was used as a control cytosolic protein. Original blots are presented in Supplementary Fig. 9.

V-ATPase transports protons across the membrane using energy from ATP hydrolysis to generate acidic conditions^22,23^. The V_1_ sector of V-ATPase protrudes from the membrane and has catalytic sites, while the V_o_ sector is embedded in the membrane and forms a proton pathway (Fig. 1A). V-ATPase activity is regulated by reversible assembly/disassembly of the V_o_ and V_1_ sectors, which depends on energy metabolism^24^. Among 13 subunits of V-ATPase, six have cell/organelle-specific isoforms, which allows structural variation in V-ATPase^25–27^. The a subunit in the V_o_ sector, which forms a proton pathway with the c ring, has four isoforms^22,25–27^. The a1, a2, and a3 isoforms are ubiquitously expressed and localize to coated-vesicles, the Golgi apparatus/early endosomes, and late endosomes/lysosomes, respectively^22,25–27^. The a4 isoform is kidney-specific^22,25–27^. Although V-ATPase is a well-known proton pump, a study of *oc*/*oc* mice, which are a osteopetrosis model with a mutation in the *TCIRG1* gene encoding the a3 isoform, revealed that a3 is important for insulin production and secretion: the a3 isoform colocalizes well with insulin in pancreatic β cells, and the blood insulin level is significantly lower in *oc*/*oc* mice than in wild-type (WT) mice when the blood glucose level is high^28^.

In addition, several lines of evidence indicate that the a subunit isoforms are involved in organelle trafficking in cooperation with trafficking regulators^29,30^. We recently demonstrated that in osteoclasts, V-ATPase containing a3 localizes to secretory lysosomes and induces their outward trafficking during differentiation by interacting with the GDP-bound forms of Rab7 and Rab27A, as well as the Rab7 guanine nucleotide exchange factor (GEF) Mon1-Ccz1, and recruiting them to secretory lysosome membranes^29,30^. Rab7 is likely activated by Mon1-Ccz1 on secretory lysosome membranes. In a3 knockout (a3KO) osteoclasts, secretory lysosomes do not move to the plasma membrane, resulting in impaired release of lysosomal enzymes essential for bone resorption^29^. a3KO mice develop osteopetrosis^31^. In kidney distal tubule cells, V-ATPase harboring a2 localizes to early endosomes containing resorbed proteins and induces their inward trafficking by binding to and recruiting trafficking regulators, namely, the small GTPase ARF6 and its GEF ARNO^32,33^. These recent findings place a spotlight on the role of V-ATPase in vesicle trafficking. From the observations described above, we hypothesized that V-ATPase is involved in regulation of insulin secretion through the role of its a subunit isoforms in organelle trafficking.

In this study, we report that both a2 and a3 isoforms specifically bound to GDP-bound form Rab27A. Outward trafficking of insulin granules upon glucose stimulation was reduced in a3 knockdown (a3KD) MIN6 cells. Interestingly, return (inward) trafficking of insulin granules in response to a shift of the glucose concentration from 20 to 2.2 mM was significantly lower and the amount of secreted insulin was higher in a2 knockdown (a2KD) cells than in control cells. Furthermore, a2 interacted with EPI64, which transforms Rab27A to its GDP-bound form. Our data suggest that a2 and a3 isoforms are essential for “back” and “forth” trafficking of insulin granules in pancreatic β cells, respectively.

## Results

### Interaction between the a subunit isoforms of V-ATPase and Rab27A variants

As described above, Rab27A is responsible for insulin secretion from pancreatic β cells in response to a high blood glucose level^13^. On the other hand, in osteoclasts, the lysosome-specific a3 isoform of V-ATPase interacts with GDP-bound form Rab27A and recruits it to secretory lysosomes, which is essential for fusion between secretory lysosome membranes and the plasma membrane^29,34^. These observations prompted us to examine whether a subunit isoforms cooperate with Rab27A in insulin secretion. We first examined interactions between a subunit isoforms and various forms of Rab27A.

We co-expressed a FLAG-tagged a isoform (a1, a2, or a3) and V5-fused Rab27A variants (WT, T23N (GDP-bound form), or Q78L (GTP-bound form)) in HEK293T cells and performed immunoprecipitation using the cell lysates and an anti-FLAG antibody. V5-fused GDP-bound form Rab27A, but not GTP-bound form was co-precipitated with FLAG-a3 (Fig. 1B, middle panel), consistent with our previous result. Interestingly, V5-fused GDP-bound form Rab27A was co-precipitated more efficiently with FLAG-a2 (Fig. 1B, middle panel). The a2 and a3 isoforms of V-ATPase may play a role in trafficking of insulin granules together with Rab27A.

The a subunit in the V_o_ sector interacts with multiple subunits in V_1_ (Fig. 1A). Next, we investigated whether the a subunit that interacts with Rab27A also interacts with the subunits in V_1_. If a precipitate prepared using FLAG-tagged GDP-bound Rab27A and an anti-FLAG antibody includes both the A subunit in V_1_ and the a subunit in V_o_, this will suggest that Rab27A interacts with the a subunit that interacts with the A subunit in V_1_. V5-a2 or V5-a3, but not the A subunit, was detected in the precipitate prepared using FLAG-Rab27A and an anti-FLAG antibody (Fig. 1C,D, lanes 1–3), suggesting that Rab27A interacts with the a subunit that does not interact with subunits in V_1_. The V_1_ sector seems to physically hinder the interaction of a2 and a3 with Rab27A. On the other hand, the A subunit and V5-Rab27A were detected in a precipitate prepared using FLAG-a subunit (Fig. 1C,D, lanes 4–6). Altogether, these results suggest that the a subunit can interact with both the A subunit and Rab27A, but not simultaneously.

Next, we examined whether expressed a2 and a3 localized to membranes and whether Rab27A interacted with membrane-embedded a isoforms or those in the soluble fraction. Immunoprecipitation was performed using the membrane and soluble fractions of HEK293T cells expressing FLAG-Rab27A and V5-a2 or V5-a3. Both a isoforms were exclusively recovered in the membrane fraction and coprecipitated with FLAG-Rab27A when an anti-FLAG antibody was used for precipitation (Fig. 1E). These results suggest that expressed a2 and a3 localize to membranes and that Rab27A interacts with membrane-embedded a isoforms. It should be mentioned that the results obtained by immunoprecipitation do not always indicate direct interactions between these proteins. Further analysis is required to know whether the interactions are direct or not.

### Back-and-forth trafficking of insulin granules in response to glucose concentration shifts

**Fig. 2:**
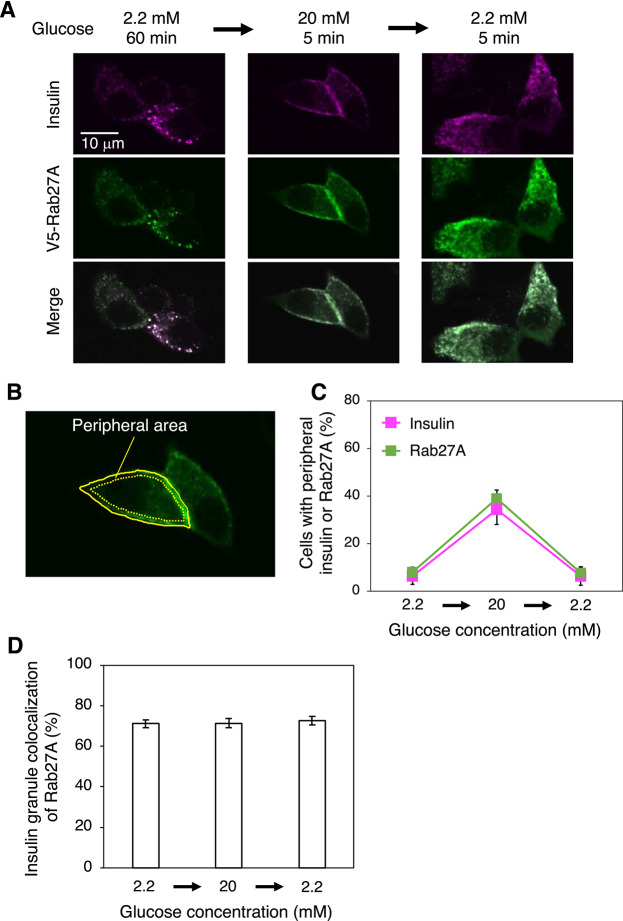
Localizations of insulin and Rab27A in the MIN6 mouse pancreatic β cell line. (**A**) Effect of the glucose concentration on localizations of insulin and V5-Rab27A. Cells expressing V5-Rab27A were sequentially incubated with 2.2 mM glucose for 60 min, 20 mM glucose for 5 min, and 2.2 mM glucose for 5 min, and then stained with antibodies specific for insulin (magenta) and V5 (green). Merged images are also shown. (**B**) Peripheral localization of insulin. The cell peripheral area was defined as the region between the cell edge (yellow solid line) and 0.75 µm inside from this edge (yellow dotted line). Cells with peripheral insulin or V5-Rab27A were defined as those in which the average signal intensity of insulin or V5-Rab27A in the cell peripheral area was more than twice that in the other area. (**C**) Cells with peripheral insulin and V5-Rab27A according to the glucose concentration. The percentages of cells with peripheral insulin (magenta) and V5-Rab27A (green) as defined in (**B**) were calculated at each glucose concentration. Data are means ± S.E. from four independent experiments. n > 30 cells in each experiment. (**D**) Localization of Rab27A to insulin granules. Co-localization was quantified as the percentage of insulin-positive pixels that were also Rab27A-positive. More than 30 cells were randomly selected from four experiments. Data are means ± S.E.

To examine the localizations of insulin and Rab27A in response to glucose concentration shifts, we expressed V5-tagged WT Rab27A in a mouse pancreatic β cell line, MIN6, which displays glucose-stimulated insulin secretion similar to that of normal islets. Immunostaining with an anti-insulin antibody revealed that insulin showed dotted signals located diffusely in the cytoplasm in cells cultured in medium containing a low concentration (2.2 mM) of glucose (Fig. 2A, magenta, left panel). After stimulation with a high concentration (20 mM) of glucose for 5 min, a significant number of insulin signals were observed at the cell periphery (Fig. 2A, magenta, middle panel). Consistent with previous studies, these observations indicate that insulin granules move toward the plasma membrane to secrete insulin upon glucose stimulation^20,35^. The peripheral localization of insulin was abrogated within 5 min when the medium was replaced by that containing 2.2 mM glucose (Fig. 2A, magenta, right panel), indicating that insulin-unreleased granules did not remain at the cell periphery but rapidly returned to the inner area after the stimulation was removed. This rapid back-and-forth trafficking of insulin granules seems important for fine-tuned control of insulin release according to glucose concentration shifts. Back-and-forth trafficking of insulin granules was also observed in MIN6 cells that did not express V5-Rab27A (Supplementary Fig. 1).

To quantify the peripheral localization of insulin, we defined the cell peripheral area as the region between the cell edge and 0.75 µm inside from this edge (Fig. 2B) and measured the fluorescence intensity in this area. We also defined cells with peripheral insulin as those in which the average signal intensity of insulin in the cell peripheral area was more than twice that in the other area. Only ~ 10% cells exhibited peripheral localization of insulin in the presence of 2.2 mM glucose (Fig. 2C, magenta). This increased to more than ~ 30% upon stimulation with 20 mM glucose for 5 min and then decreased back to ~ 10% within 5 min after the stimulation was removed.

Similar to insulin, V5-Rab27A exhibited a transient peripheral localization upon glucose stimulation (Fig. 2A,C, green). Co-localization of insulin and Rab27A was quantified as the percentage of insulin-positive pixels that were also Rab27A-positive. V5-Rab27A co-localized well with insulin under all glucose concentrations examined (Fig. 2D). These results suggest that Rab27A plays a role in both back-and-forth trafficking of insulin granules. On the other hand, it is unlikely that Rab27A recruitment to insulin granules triggers their trafficking because Rab27A localized to insulin granules even under low glucose conditions.

### Effects of a2 on back-and-forth trafficking of insulin granules

**Fig. 3:**
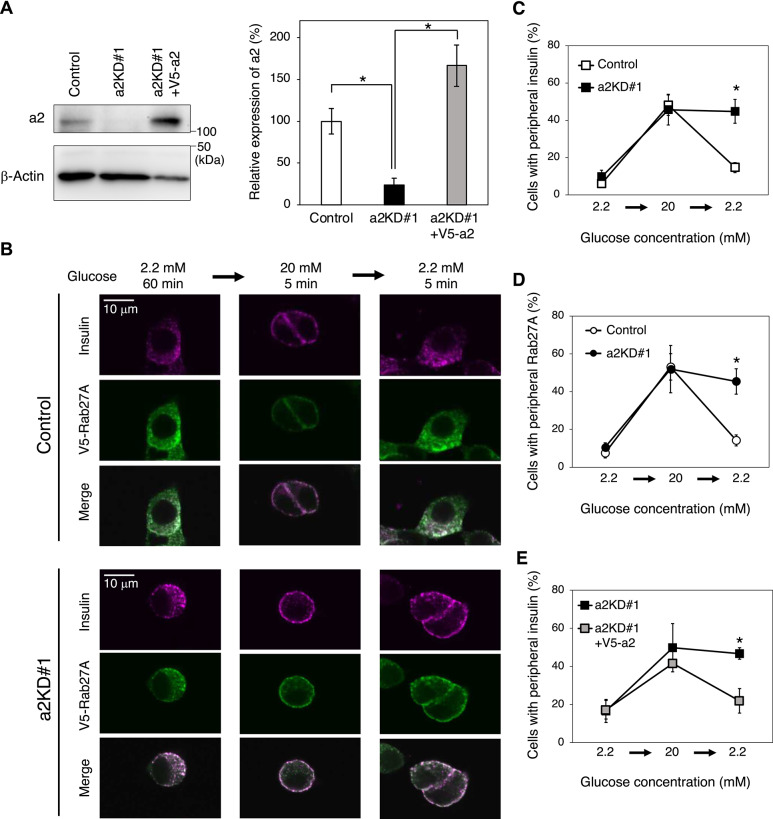
Localization of insulin granules in a2KD MIN6 cells. (**A**) Expression of a2 in a2KD#1 cells. MIN6 cells were transduced with pSIREN-RetroQ-negative control shRNA (control) or pSIREN-RetroQ-a2-shRNA#1 (a2KD#1). a2KD#1 cells were further transduced with pMX-V5-a2 (a2KD#1 + V5-a2). Endogenous a2 and V5-a2 were detected with an antibody specific for a2 (left upper panel). β-actin was also detected using a corresponding antibody (left lower panel). Original blots are presented in Supplementary Fig. 10. The blots were cut horizontally at ~ 75 kDa. Upper and lower membranes were subjected to hybridization with anti-a2 and anti-β-actin antibodies, respectively. A full-length membrane was cut to conserve the anti-a2 antibody, which is commercially unavailable. Relative signal intensities of a2 were calculated as means ± S.E. from three independent experiments compared with control cells (set to 100%) (right panel). (**B**) Localizations of insulin and V5-Rab27A in control (upper panels) and a2KD#1 (lower panels) cells. Cells expressing V5-Rab27A were sequentially incubated with 2.2 mM glucose for 60 min, 20 mM glucose for 5 min, and 2.2 mM glucose for 5 min, and then stained with antibodies specific for insulin (magenta) and V5 (green). Merged images are also shown. (**C**–**E**) Cells with peripheral insulin and Rab27A according to the glucose concentration. The percentage of cells with peripheral insulin (**C**, **E**) or Rab27A (**D**) was calculated as described in Fig. 2B. Control cells, open squares (insulin), open circles (Rab27A); a2KD#1 cells, closed squares (insulin), closed circles (Rab27A); a2KD#1 + V5-a2 cells, shaded squares (insulin). Data are means ± S.E. from more than three independent experiments. n > 30 cells in each experiment. *p < 0.05, (**A**) unpaired two-tailed Student’s t-test, (**C**–**E**) Unpaired multiple t test (Holm-Šídák method).

We examined the effects of a2 on back-and-forth trafficking of insulin granules upon glucose stimulation using the experimental system shown in Fig. 2. In a2KD#1 MIN6 cells, expression of a2 was ~ 20% of that in cells expressing control shRNA (Fig. 3A, a2KD#1). On the other hand, when V5-fused a2 was expressed in a2KD#1 cells, expression of a2 increased to ~ 150% of that in control cells (Fig. 3A, a2KD#1 + V5-a2). Immunostaining of insulin and V5-Rab27A demonstrated that these proteins clearly co-localized in a2KD#1 cells similar to control cells (Fig. 3B, magenta and green, respectively). These proteins exhibited a peripheral localization upon glucose stimulation both in control and a2KD#1 cells (Fig. 3B, left and middle panels). This peripheral localization was abrogated in control cells, but not in a2KD#1 cells, when the glucose concentration was shifted from 20 to 2.2 mM (Fig. 3B, right panels). Quantitative analysis of insulin and Rab27A localization showed that outward trafficking of insulin granules with Rab27A in a2KD#1 cells was similar to that in control cells, but inward trafficking of insulin granules upon removal of the stimulation was hardly observed (Fig. 3C,D). Similar results were obtained using a2KD#2 MIN6 cells transduced with a2 shRNA#2 (Supplementary Fig. 2). Furthermore, inward trafficking of insulin granules was recovered in a2KD#1 cells expressing V5-fused a2 (Fig. 3E, and Supplementary Fig. 3). These results indicate that a2 is involved in back (inward) trafficking of insulin granules after glucose stimulation is removed.

### Involvement of microtubules in back trafficking of insulin granules

In general, turn-off of insulin secretion has been considered passively regulated, depending on attenuation of the secretion signal. On the other hand, our findings that Rab27A remains on insulin granules even after glucose stimulation is removed and that a2 is required for inward movement of insulin granules indicate that this movement occurs actively. Therefore, we examined whether microtubules are required for inward movement of insulin granules. If the granules just passively defuse in the cytosol after the stimulation is removed, microtubules should not be necessary.

**Fig. 4:**
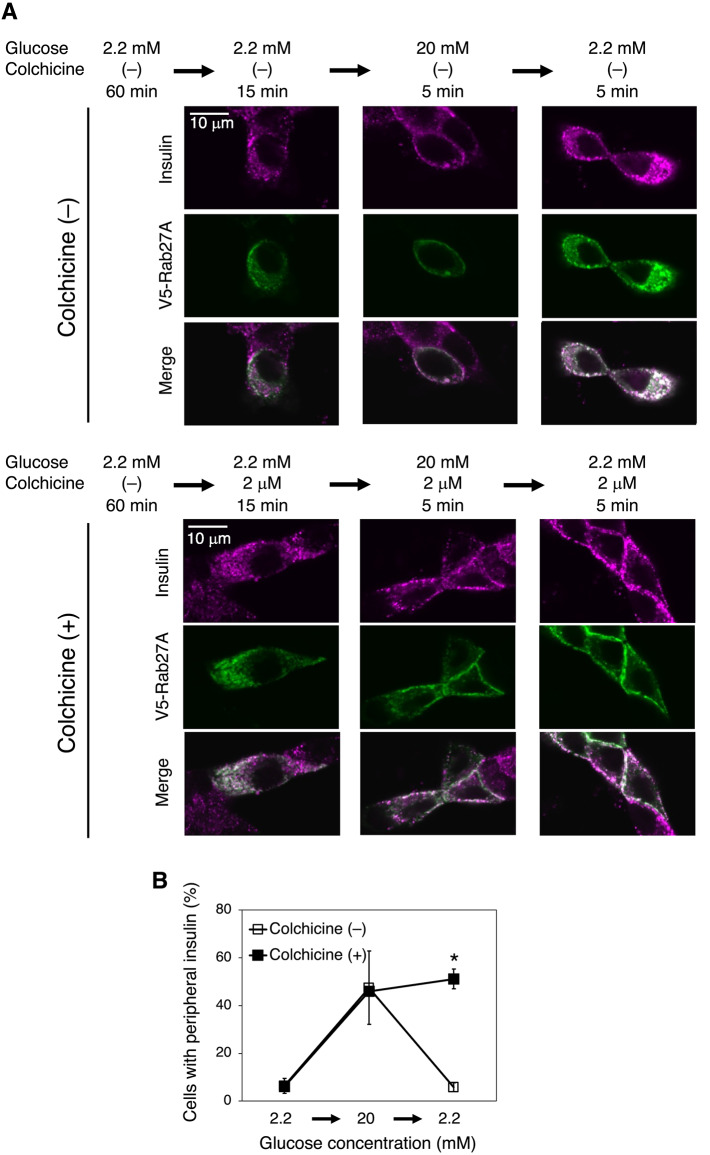
Effects of colchicine on localizations of insulin and Rab27A in MIN6 cells. (**A**) Localizations of insulin and V5-Rab27A in cells treated with colchicine. Cells expressing V5-Rab27A were preincubated with 2.2 mM glucose for 60 min, and then, in the absence (colchicine (–), upper panels) or presence (colchicine ( +), lower panels) of 2 μM colchicine, sequentially incubated with 2.2 mM glucose for 15 min, 20 mM glucose for 5 min, and 2.2 mM glucose for 5 min. The treated cells were stained with antibodies specific for insulin (magenta) and V5 (green). Merged images are also shown. (**B**) Effects of colchicine on the glucose-dependent peripheral localization of insulin. The percentage of cells with peripheral insulin was calculated in the absence (colchicine (–), open squares) and presence (colchicine ( +), closed squares) of 2 μM colchicine. Data are means ± S.E. from more than three independent experiments. n > 30 cells in each experiment. *p < 0.05, unpaired multiple t test (Holm-Šídák method).

Cells expressing V5-Rab27A were treated with colchicine, an inhibitor of tubulin polymerization. Microtubules were observed in the absence of colchicine (Supplementary Fig. 4, colchicine (–), magenta) but not in cells treated with colchicine (Supplementary Fig. 4, colchicine ( +)). In these cells, the localizations of insulin and V5-Rab27A were investigated upon glucose concentration shifts. Insulin and V5-Rab27A co-localized well under all conditions and moved to the cell periphery upon glucose stimulation in both colchicine-treated and untreated cells (Fig. 4A, left and middle panels, and Fig. 4B). However, unlike in colchicine-untreated cells, the peripheral localization was not abolished in colchicine-treated cells after the stimulation was removed (Fig. 4A, right panels, and Fig. 4B, closed squares). These results indicated that microtubules were essential for inward movement of insulin granules. Taken together, these findings show that inward movement of insulin granules after removal of the stimulation is not passive, but actively regulated by microtubules and a2.

As described above, outward trafficking of insulin granules was observed upon glucose stimulation and colchicine treatment (Fig. 4A, left and middle panels, and Fig. 4B). This is probably because actin filaments are involved in outward trafficking of insulin granules^36^.

### Effects of a2 on insulin secretion and insulin granule morphology

**Fig. 5:**
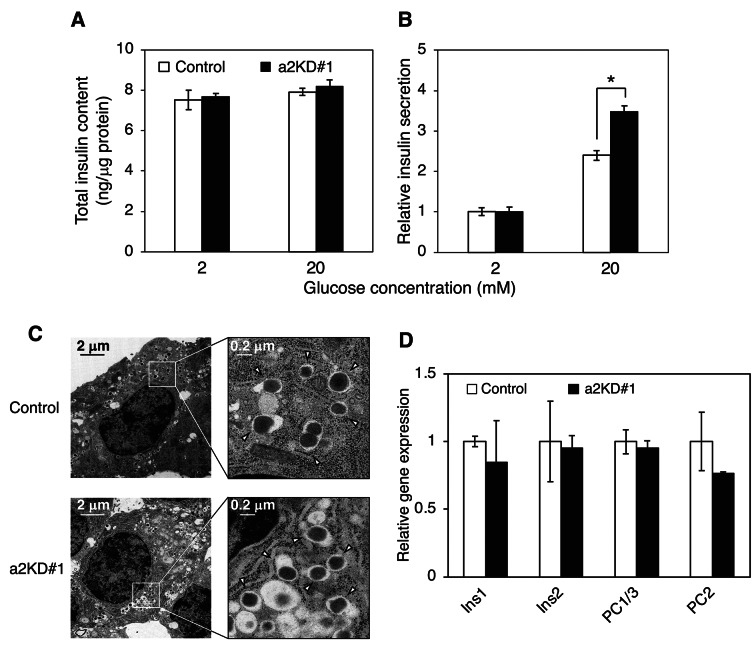
Insulin secretion, morphology of insulin granules, and expression of insulin and its related genes in control and a2KD MIN6 cells. (**A**) Total insulin content in control (open bars) and a2KD#1 (closed bars) cells. (**B**) Insulin secretion from control (open bars) and a2KD#1 (closed bars) cells. Cells were treated with KRH buffer containing 2 or 20 mM glucose for 2 h, and then the insulin content in the recovered medium and acid ethanol extracts was determined using an ELISA kit. Insulin secretion from control cells treated with 2 mM glucose was set to 1. Data are means ± S.E. from four independent experiments. (**C**) Electron micrographs of control (upper panels) and a2KD#1 (lower panels) cells in HEPES-buffered Krebs buffer containing 2.2 mM glucose. Magnified images of the boxed areas are shown in the right panels. Arrows indicate typical insulin granules with a dense core. (**D**) Expression of insulin and insulin-related genes in control (open bars) and a2KD#1 (closed bars) cells. Total mRNA was isolated from cells in HEPES-buffered Krebs buffer containing 2.2 mM glucose. The expression levels of *Ins1*, *Ins2*, *PC1/3*, and *PC2* were quantified by real-time RT-PCR using *β-actin* as an internal standard, with expression in control cells set to 1. *Ins1*, insulin 1; *Ins2*, insulin 2; *PC1/3*, prohormone convertase 1/3; *PC2*, prohormone convertase 2. Data are means ± S.E. from three independent experiments. *p < 0.05, unpaired two-tailed Student’s t-test.

Next, we investigated the effects of a2 on insulin secretion. The total insulin content was ~ 7 ng/μg protein and was similar in control and a2KD#1 cells and in low (2 mM) and high (20 mM) glucose conditions (Fig. 5A). The amount of insulin secreted into the medium was measured using an insulin ELISA kit after incubation in 2 or 20 mM glucose-containing medium for 2 h. High glucose treatment increased the amount of secreted insulin ~ 2.5-fold in control cells (Fig. 5B, open bars) and ~ 3.5-fold in a2KD#1 cells (Fig. 5B, closed bars), indicating that insulin secretion was promoted in a2KD#1 cells. A similar tendency was observed in a study using a2 knockout (a2KO) mice^37^. More insulin granules stayed at the periphery after glucose stimulation was removed in a2KD#1 cells (Fig. 3). Thus, uncontrolled insulin release likely occurred in these cells. The a2 isoform seems to be involved in rapid cessation of insulin release after the stimulation is removed via its function in inward trafficking of insulin granules.

Next, we checked the morphology of insulin granules in control and a2KD#1 cells. Electron microscopy revealed that insulin granules in these cells were indistinguishable in terms of size, shape, and insulin density (Fig. 5C), indicating that increased insulin secretion in a2KD#1 cells is not due to a defect in insulin granules. We also performed real-time PCR to test gene expression of insulin (*ins1* and *ins2*) and proinsulin-converting enzymes (*PC1/3* and *PC2*), which are required for insulin maturation. Expression of these genes was similar in control and a2KD#1 cells (Fig. 5D and Supplementary Fig. 5, open and closed bars, respectively), suggesting that increased insulin secretion in a2KD#1 cells is not due to a change in gene expression or maturation of insulin.

### Interaction between a2 and the Rab27A GAP EPI64

**Fig. 6:**
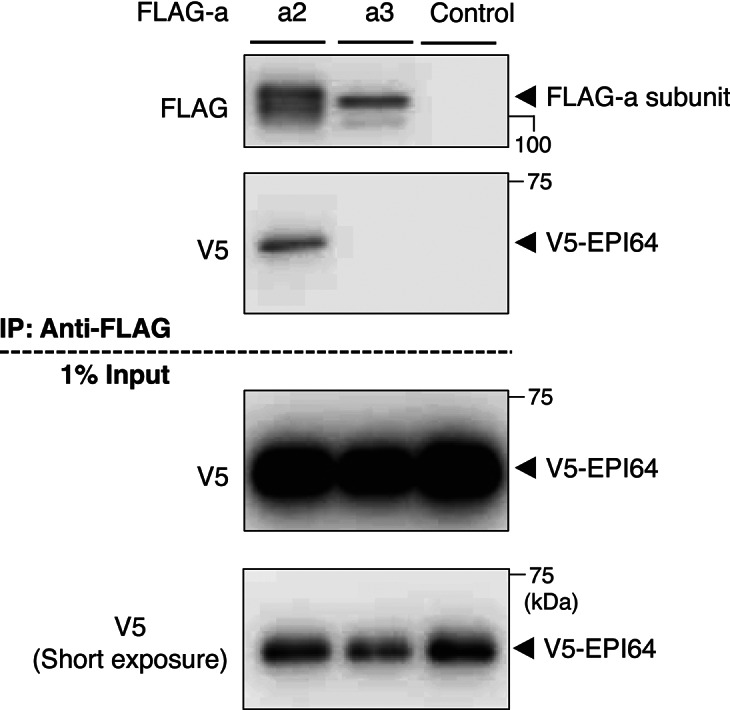
Interaction of a2 and a3 with EPI64, the GAP for Rab27A. FLAG-a2 or FLAG-a3 and V5-EPI64 were co-expressed in HEK293T cells. The cells were lysed, and lysates were immunoprecipitated with an anti-FLAG antibody. The precipitates were analyzed using antibodies against FLAG (top panel) and V5 (second panel from the top). In addition, 1% of the cell lysate was subjected to western blotting with an anti-V5 antibody (bottom two panels). Original blots are presented in Supplementary Fig. 11.

Rab27A localized to insulin granules independent of glucose stimulation (Fig. 2), suggesting that it was almost always GTP-bound (active form), remained on membranes of insulin granules, and was involved in both back-and-forth trafficking of insulin granules. The directions of back-and-forth trafficking are opposite; therefore, Rab27A should transiently become GDP-bound (inactive form) at the cell periphery to exchange its effectors from outward-specific to inward-specific versions. Recently, it was reported that the Rab27A GTPase-activating protein (GAP) EPI64 converts Rab27A from the GTP-bound to the GDP-bound form at the plasma membrane^21,38^. a2 interacts with GDP-bound form Rab27A; therefore, we examined the interaction between a2 and EPI64. FLAG-a2 and V5-EPI64 were co-expressed in HEK293T cells, and immunoprecipitation was performed using an anti-FLAG antibody. As expected, V5-EPI64 was co-precipitated with FLAG-a2, indicating that a2 interacted with EPI64 (Fig. 6). Interestingly, V5-EPI64 was not co-precipitated with FLAG-a3, indicating that EPI64 preferentially binds to a2 over a3 (Fig. 6). These results suggest that a2 and a3 employ different molecules for insulin vesicle trafficking and are consistent with our claim that a2 and a3 play different roles in insulin granule trafficking.

### Effects of a3 on back-and-forth trafficking of insulin granules

**Fig. 7:**
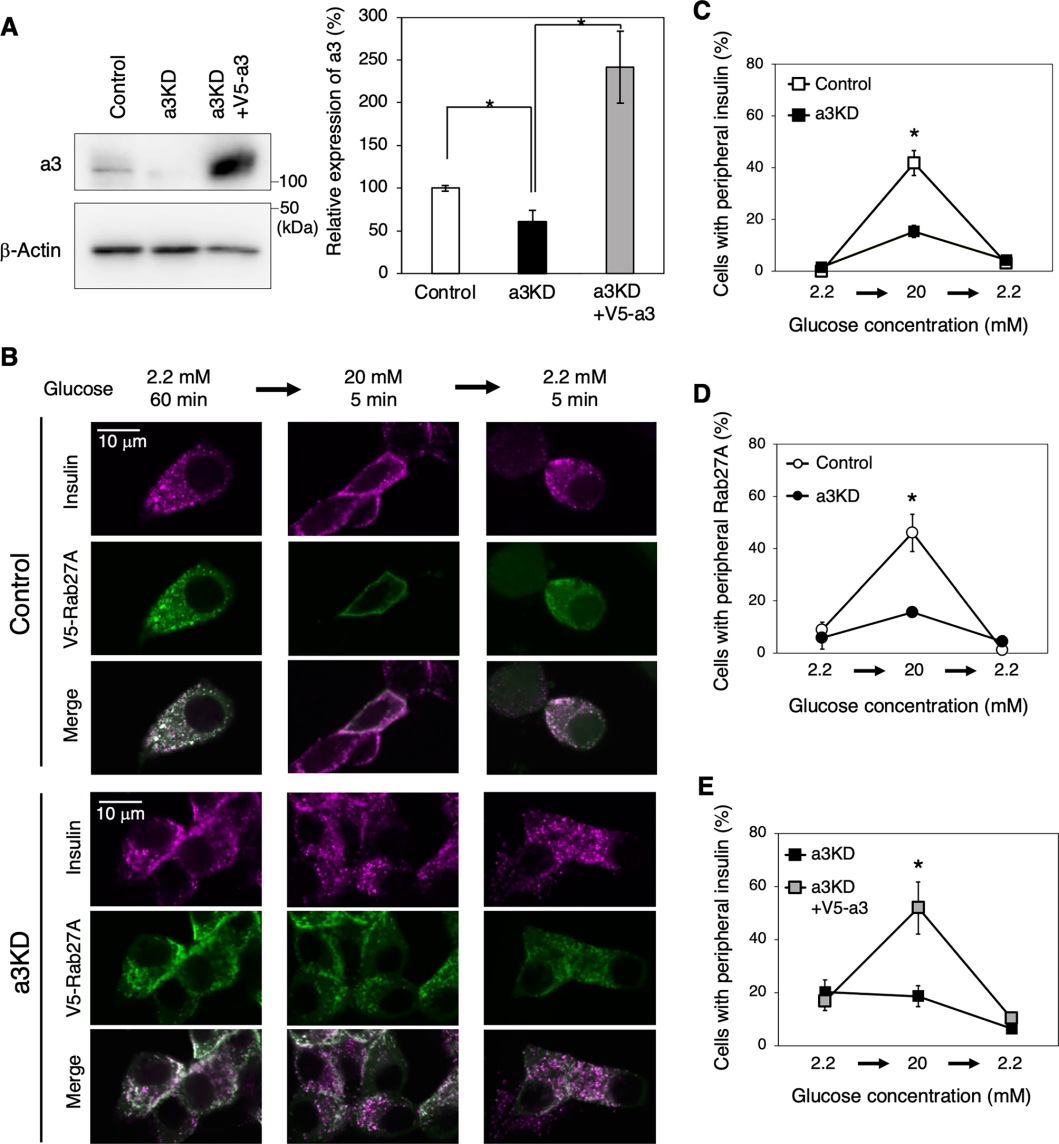
Localization of insulin granules in control and a3KD MIN6 cells. (**A**) Expression of a3 in a3KD cells. Cells were transduced with pSIREN-RetroQ-negative control shRNA (control) or pSIREN-RetroQ-a3-shRNA (a3KD). a3KD cells were further transduced with pMX-V5-a3 (a3KD + V5-a3). Endogenous a3 and V5-a3 were detected with an antibody specific for a3 (left upper panel). β-actin was also detected using a corresponding antibody (left lower panel). Original blots are presented in Supplementary Fig. 12. Relative signal intensities of a3 were calculated as means ± S.E. from three independent experiments compared with control cells (set to 100%) (right panel). (**B**) Localizations of insulin and V5-Rab27A in control (upper panels) and a3KD (lower panels) cells. Cells expressing V5-Rab27A were sequentially incubated with 2.2 mM glucose for 60 min, 20 mM glucose for 5 min, and 2.2 mM glucose for 5 min, and then stained with antibodies specific for insulin (magenta) and V5 (green). Merged images are also shown. (**C**–**E**) Cells with peripheral insulin and Rab27A according to the glucose concentration. The percentage of cells with peripheral insulin (**C**, **E**) or Rab27A (**D**) was calculated as described in Fig. 2B. Control cells, open squares (insulin), open circles (Rab27A); a3KD cells, closed squares (insulin), closed circles (Rab27A); a3KD#1 + V5-a3 cells, shaded squares (insulin). Data are means ± S.E. from three independent experiments. n > 30 cells in each experiment. *p < 0.05, (**A**) unpaired two-tailed Student’s t-test, (**C**–**E**) Unpaired multiple t test (Holm-Šídák method).

a3 localizes to insulin granules in mouse and has been suggested to play a role in insulin secretion^28^, but the molecular mechanism is unknown. We expected a3 to be involved in forth (outward) trafficking of insulin granules in MIN6 cells. To investigate this, we prepared a3KD MIN6 cells and examined the effect of a3 on insulin granule trafficking. In a3KD cells, a3 expression was decreased to ~ 50% of that in control cells (Fig. 7A, Control and a3KD). When V5-a3 was expressed in a3KD cells, a3 expression increased to ~ 250% of that in control cells (Fig. 7A, Control and a3KD + V5-a3). As expected, the peripheral localization of insulin and Rab27A in response to glucose stimulation was significantly lower in a3KD cells than in control cells (Fig. 7B, left and middle panels, and 7C, D). On the other hand, the peripheral localization of insulin was recovered in a3KD cells expressing V5-a3 (Fig. 7E and Supplementary Fig. 6). These results indicate that a3 is involved in outward trafficking of insulin granules.

**Fig. 8:**
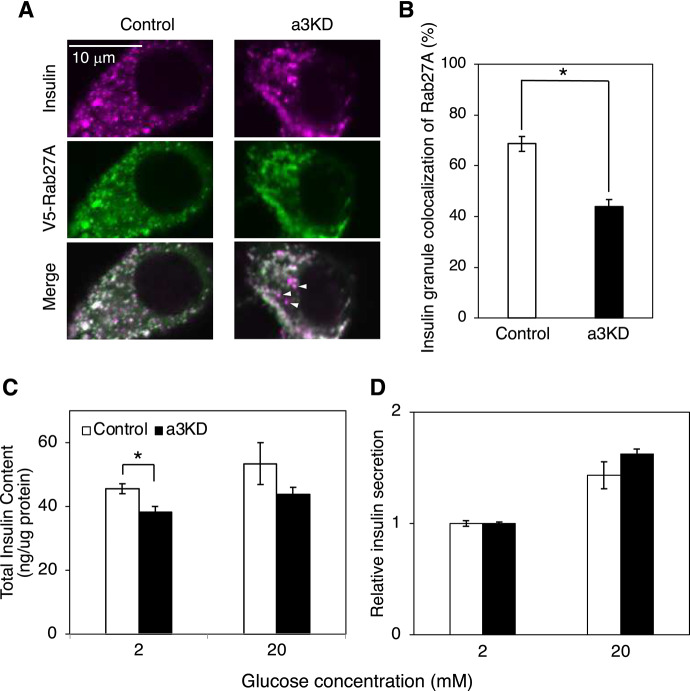
Localization of Rab27A to insulin granules and insulin secretion in control and a3KD MIN6 cells. (**A**) Effect of a3 on the localization of Rab27A to insulin granules. Control and a3KD cells expressing V5-Rab27A were incubated with 2.2 mM glucose for 60 min and stained with antibodies specific for insulin (magenta) and V5 (green). Merged images are also shown. (**B**) Co-localization was quantified as the percentage of insulin-positive pixels that were also Rab27A-positive. Open and closed bars indicate control and a3KD cells, respectively. More than 30 cells were randomly selected from three experiments using WT and a3KD cells. Data are means ± S.E. (**C**) Total insulin content in control (open bars) and a3KD (closed bars) cells. (**D**) Insulin secretion from control (open bars) and a3KD (closed bars) cells. Cells were treated with KRH buffer containing 2 or 20 mM glucose for 2 h, and then the insulin content in the recovered medium and acid ethanol extracts was determined using an ELISA kit. Insulin secretion from control cells treated with 2 mM glucose was set to 1. Data are means ± S.E. from four independent experiments. *p < 0.05, unpaired two-tailed Student’s t-test.

If a3 recruits Rab27A to insulin granules like it recruits Rab7 to secretory lysosomes in osteoclasts^29^, the localization of Rab27A to insulin granules will be decreased in a3KD MIN6 cells. Immunostaining of MIN6 cells expressing V5-Rab27A revealed that V5-Rab27A partly co-localized with insulin in a3KD cells, but this co-localization was lower than that in control cells (Fig. 8A, right panels, arrowheads). The percentage of insulin-positive pixels that were also V5-Rab27A-positive was significantly lower in a3KD cells (~ 40%) than in control cells (~ 70%) (Fig. 8B). This suggests that a3 is important for localization of Rab27A to insulin granules.

Next, we examined the effects of a3 on insulin secretion in the same way as was used for a2. The total insulin content was slightly decreased in a3KD cells (Fig. 8C), consistent with a previous observation using a3-deficient mice^28^. However, in contrast with the decreased insulin secretion in a3-deficient mice, insulin secretion in a3KD MIN6 cells was almost the same as that in control cells (Fig. 8D). This discrepancy may be due to incomplete suppression of outward trafficking of insulin granules in a3KD cells (Fig. 7C).

Further investigations are required to fully elucidate the role of a3 in outward trafficking of insulin granules.

## Discussion

This is the first report to show that a subunit isoforms of the proton pump V-ATPase are responsible for insulin granule trafficking that is important for insulin secretion in a pancreatic β cell line. Specifically, the a2 and a3 isoforms of V-ATPase are involved in glucose concentration shift-induced back-and-forth trafficking of insulin granules, respectively, probably via their interactions with GDP-bound Rab27A. The molecular mechanism we elucidated is unique: insulin granules are transported in opposite directions according to glucose concentration shifts dependent on a subunit isoforms of V-ATPase.

Insulin granules moved toward the plasma membrane upon glucose stimulation, but most did not release insulin and rapidly returned to the inner area after glucose stimulation was removed. This is consistent with the previous finding that less than 15% of insulin (relative to the total) is released from MIN6 cells upon any glucose stimulation^11^. When a2 was depleted, rapid back trafficking of insulin granules was not observed and insulin secretion was promoted. Therefore, rapid back trafficking is likely important to prevent uncontrolled insulin release at the plasma membrane and to promptly stop insulin release after the blood glucose level returns to normal. The events that occur after glucose stimulation have not received sufficient attention, and actively regulated inward trafficking of insulin granules should be further studied to elucidate the detailed mechanism underlying fine-tuned regulation of insulin secretion.

Rab family proteins are regulated by guanine nucleotides: the GTP-bound form is active and localizes to membranes, while the GDP-bound form is inactive and stays in the cytoplasm^39^. In the case of Rab27A, the GAP EPI64 promotes its GTPase activity and converts it to the GDP-bound form^21,38^. GDP-bound form Rab27A is unstable and most Rab27A is GTP-bound in various cells^40,41^. Consistently, Rab27A always localized to insulin granules in MIN6 cells independent of the glucose concentration, suggesting that it plays a role in both back-and-forth trafficking of insulin granules. The direction of organelle trafficking depends on the effectors connected to Rab proteins; therefore, Rab27A should be transiently inactive at the cell periphery to dissociate from its outward-specific effectors and associate with its inward-specific effectors in response to a glucose concentration shift from high to low. EPI64 was recently shown to localize to the plasma membrane in a glucose-dependent manner^21,38^. a2 interacted with both GDP-bound form Rab27A and EPI64, and is therefore likely involved in Rab27A inactivation by EPI64. It is possible that a2 interacts with and thereby transiently stabilizes GDP-bound Rab27A at the cell periphery.

To elucidate the detailed mechanism, it is important to determine the localization of a2 in β cells. a2 mainly localizes to the Golgi apparatus in various cell types^42–44^. By immunostaining MIN6 cells expressing V5-a2, we detected a2 in the Golgi apparatus but could not detect it in insulin granules. However, these results do not mean that a2 is always absent from organelles other than the Golgi apparatus. For example, a2 is detected in early endosomes in proximal tubule cells^32^. Furthermore, a2 is strongly detected in insulin-positive granules in a3-deficient mice^28^, indicating there is a system to transport a2 to insulin granules. Additionally, an undetectable level of a2 can induce secretory lysosome trafficking in osteoclasts^45^, suggesting that a2 is functional even when it is not detected in an organelle by immunostaining. Therefore, we predict that an undetectable level of a2 localizes to insulin granules and plays a role in Rab27A regulation; however, a further study is required to determine the localization of a2 in β cells and to elucidate the molecular mechanism underlying trafficking of insulin granules involving a2.

On the other hand, a3 localizes to insulin granules in mouse pancreatic β cells^28^. In this study, we showed that a3 is involved in outward trafficking of insulin granules upon glucose stimulation, interacts with GDP-bound form Rab27A, and is important for localization of Rab27A to insulin granules. What is the role of a3 in outward trafficking of insulin granules? In osteoclasts, a3 localizes to secretory lysosomes and recruits trafficking regulators, such as Rab7, Rab27A, and the Rab7 GEF Mon1-Ccz1, to them^29^. a3 specifically interacts with GDP-bound forms of Rab7 and Rab27A, and these inactive Rab proteins recruited by a3 are probably activated by their GEFs on secretory lysosomes. Recruitment and activation of Rab7 trigger outward trafficking of secretory lysosomes in osteoclasts^29^. Considering the similarity with its function in osteoclasts, a3 likely recruits GDP-bound Rab27A to insulin granules. Thereafter, Rab27A needs to be activated by its GEF for its stable localization to insulin granules, which is essential for their outward trafficking. However, unlike secretory lysosomes in osteoclasts, recruitment of Rab27A does not trigger outward trafficking of insulin granules because Rab27A localizes to these granules in a glucose-independent manner.

Our data indicate that Rab27A interacts with the a subunit in V_1_-dissociated V-ATPase. Interestingly, glucose depletion promotes dissociation of V_1_ from V_o_^24^. Based on these observations, we propose the following scheme for recruitment of Rab27A to insulin granules. Before glucose stimulation, V_1_ is dissociated from V_o_, which enables a3 to interact with and recruit GDP-bound Rab27A to insulin granules. Then, Rab27A is activated by its GEF, released from a3, and stably localizes to the membrane of granules. When the glucose concentration increases, V_1_ associates with V_o_, which may inhibit the interaction between a3 and GDP-bound Rab27, but insulin granule trafficking is unaffected because activated Rab27A is already localized to the granule membrane. This scheme is consistent with the glucose-independent localization of Rab27A to insulin granules. A further study is essential to fully elucidate the molecular mechanism underlying back-and-forth trafficking of insulin granules involving a2 and a3.

Previous studies together with ours demonstrate that a2 is involved in inward trafficking of early endosomes in the kidneys and of insulin granules in pancreatic β cells, while a3 is involved in outward trafficking of secretory lysosomes in osteoclasts and insulin granules^28,29,32^. In kidney proximal tubule epithelial cells, the small GTPase Arf6 and its GEF, ARNO (ADP-ribosylation factor nucleotide site opener), interact with the c subunit and a2 isoform of V-ATPase in early endosomes, respectively, which is essential for inward trafficking of early endosomes^32^. These interactions require luminal acidification of early endosomes by V-ATPase. As described above, in osteoclasts, a3 in secretory lysosomes interacts with and recruits Rab7 and its GEF, Mon1-Ccz1, which is indispensable for outward trafficking of secretory lysosomes^30^. Luminal acidification by V-ATPase is also required for this outward trafficking^46^. On the other hand, luminal acidification is not required for insulin secretion^28^. These observations reveal significant similarities in the role of V-ATPase in organelle/vesicle trafficking, but it is noteworthy that there is a discrepancy in the importance of luminal acidification by V-ATPase for this trafficking.

The a subunit isoforms of V-ATPase are likely a determinant of the direction of organelle trafficking. These isoforms may play a similar role in other types of organelle trafficking in other cells, such as melanin secretion from melanocytes, neurotransmitter secretion from neurons, and perforin secretion from cytotoxic T cells. The a subunit isoforms of V-ATPase seem to be indispensable for characteristic organelle trafficking in various cells.

## Methods

### Cell culture

HEK293T and Plat-E cells were purchased from the RIKEN BioResource Research Center (RCB2202) and Cell Biolab, respectively. MIN6 cells were kindly donated by Prof. Junichi Miyazaki (Osaka University)^47^. HEK293T and Plat-E cells were cultured in Dulbecco’s modified Eagle’s medium (DMEM, high glucose, pyruvate; Gibco, 11995065) containing 10% fetal bovine serum (Gibco, 10270106), 100 units/mL penicillin, and 100 μg/mL streptomycin. The medium used to culture Plat-E cells also contained 10 μg/mL blasticidin S (Kaken Pharmaceuticals, KK-400) and 1 μg/mL puromycin (Santa Cruz, sc-108071). MIN6 cells were cultured in DMEM without pyruvate (DMEM, high glucose, no glutamine; Gibco, 11960044) containing 15% fetal bovine serum, 292 μg/mL glutamate, 100 units/mL penicillin, and 100 μg/mL streptomycin. The medium used to culture all cell lines was changed every third or fourth day, and the cells were maintained at 37 °C in a humidified atmosphere containing 5% CO_2_. The cells were grown in 10 cm dishes (Thermo Scientific, 150467) and passaged using 0.25% trypsin/EDTA solution (Gibco, 25200056) when they reached 90% confluency. Unless otherwise indicated, all reagents used for cell culture were from Thermo Scientific.

### Construction of plasmids

FLAG-a2 and -a3 and V5-a2, -a3, and -Rab27A (WT, T23N, and Q78L) in pcDNA3.1 were constructed previously^29^. For retrovirus infection, V5-a2 and -a3 were subcloned from pMX(puro) into pMX(neo) (Cell Biolabs, RTV-011) using the *Eco*RI and *Not*I sites^45^. To construct V5-EPI64 in pcDNA3.1, DNA fragments of EPI64 was obtained by reverse transcriptase-polymerase chain reaction (RT-PCR) using total RNA isolated from MIN6 cells. Total RNA was extracted using an RNeasy Micro Kit (Qiagen, 74004). The DNA fragment of EPI64 was ligated into pcDNA3.1 using the *Bam*HI and *Not*I sites. To construct control, a2KD#1, a2KD#2, and a3KD plasmids, synthesized oligonucleotides encoding negative control, a2KD#1, a2KD#2, and a3KD shRNAs were annealed and ligated into pSIREN-RetroQ (Takara, 631526) using the *Bam*HI and *Eco*RI sites. The oligonucleotides used in this study are listed in Supplementary Table 1.

### Retrovirus infection

For retrovirus production, 1 × 10^6^ Plat-E packaging cells (Cell Biolab) were plated in a 60 mm dish (Thermo Scientific, 150,462). After incubation for 24 h, the cells were transfected with 2.5 μg of pMX and pSIREN-RetroQ retroviral vectors using XtremeGENE9 (Roche Diagnostics, XTG9). After incubation for 48 h, the culture medium containing the recombinant virus was harvested and filtered using a 0.45 μm syringe filter (Millipore, SLHVR33RS). MIN6 cells were infected with the virus in the presence of 2.5 μg/mL polybrene. After incubation for 24 h, the medium was changed to that containing 5 μg/mL puromycin or 1 mg/mL G418 (Roche Diagnostics, G418) and the cells were cultured for 48 h to select infected cells.

### Antibodies

Antibodies against V5 were purchased from Life Technologies (R96025), Abcam (ab206573), and Cell Signaling (D3H8Q). Antibodies against α-tubulin (DM1A, T9026), β-actin (AC-15, A5441), and FLAG (F7425) were purchased from Sigma-Aldrich. Antibodies against a2 and a3 were generated as described previously^48^. Alexa Fluor-conjugated secondary antibodies (A11006, A11029, A11030, A11034, A11081, and A21236) were purchased from Life Technologies. Horseradish peroxidase-conjugated antibodies against rabbit IgG (NA934VS), mouse IgG (NA931VS), and chicken IgY (12–341) were purchased from GE Healthcare (anti-rabbit IgG and -mouse IgG) and Millipore (anti-chicken IgY). Clean-Blot, an HRP-conjugated antibody for post-immunoprecipitation western blot detection of native primary antibodies, was purchased from Thermo Scientific.

### Immunoprecipitation

Immunoprecipitation was performed as described previously^29^. Briefly, 0.5 × 10^6^ HEK293T cells were plated in a 60 mm dish. After incubation for 24 h, the cells were transfected with 2.5 μg of expression plasmids using XtremeGENE9. At 48 h post-transfection, the cells were lysed in 1 mL of immunoprecipitation buffer (1% Triton X-100, 10% glycerol, 50 mM Tris–HCl pH 7.4, 150 mM NaCl, 1 mM dithiothreitol, 1 mM EDTA, 1 mM phenylmethanesulfonyl fluoride, and a protease inhibitor cocktail). The lysate was centrifuged for 10 min at 18,300 × g at 4 °C, and the supernatant (input) was recovered and mixed with anti-FLAG M2-agarose (Sigma-Aldrich, A2220). After incubation for 2 h at 4 °C, the immunoprecipitate was collected by centrifugation, washed three times with immunoprecipitation buffer, and analyzed by western blotting.

For subcellular fractionation, at 48 h post-transfection, cells were disrupted in 0.45 mL of 0.25 M sucrose containing 3 mM imidazole (pH 7.4), 0.5 mM EDTA, and 1 mM phenylmethanesulfonyl fluoride, and centrifuged at 750 × g for 5 min. The resulting post-nuclear supernatant was centrifuged at 100,000 × g for 30 min, and membrane and soluble fractions were obtained. After addition of immunoprecipitation buffer up to 1 mL, these fractions were immunoprecipitated as described above.

### Western blotting

MIN6 cells were lysed in SDS–polyacrylamide gel electrophoresis (SDS-PAGE) sample buffer (62.5 mM Tris-HCl pH 6.8, 1% sodium dodecyl sulphate, 5% glycerol, and 5% 2-mercaptoethanol). The total protein concentrations of the samples were quantified using XL-Bradford (Aproscience, KY-1030). SDS-PAGE sample buffer was added to inputs and immunoprecipitates from HEK293 cells. The samples were boiled at 70 °C for 5 min, separated by 10.5% SDS-PAGE, and transferred to polyvinylidene fluoride membranes. The membranes were blocked with 5% skim milk prepared in phosphate-buffered saline containing 0.05% Tween-20 (PBS-T) for 1 h at room temperature. Subsequently, the membranes were probed with primary antibodies against FLAG (1:1000), V5 (1:500), α-tubulin (1:1000), β-actin (1:1500), a2 (1:500), a3 (1:500), and A (1:500), which were diluted in PBS-T containing 5% skim milk, overnight at 4 °C. The membranes were washed three times with PBS-T and then probed with horseradish peroxidase-conjugated antibodies against rabbit IgG (1:1000), mouse IgG (1:1000), or chicken IgY (1:1000) or Clean Blot (1:1000, Thermo Scientific) as secondary antibodies for 1 h at room temperature. Immune complexes were detected by chemiluminescence using an ECL Prime Detection Kit (GE Healthcare, GERPN2236) and a LAS-3000 (FUJIFILM) or FUSION Solo 7S (Viber) imaging system.

### Glucose stimulation and fluorescence microscopy

A total of 1 × 10^6^ MIN6 cells in a 2-mm gap electroporation cuvette (Nepa Gene, EC-002S) were transfected with 2 μg of expression plasmids by electroporation using CUY21Pro-Vitro (Nepa Gene). The conditions for electroporation were as follows: a poring pulse of 175 V (P on: 7.5 ms, P off: 50 ms) and a driving pulse of 20 V (P on: 50 ms, P off: 50 ms, ten cycles). Transfected cells were plated at a density of 2.5 × 10^5^ cells/well into 24-well plates (Thermo Scientific, 3524) containing autoclaved glass coverslips. At 72 h post-transfection, cells were preincubated with HEPES-buffered Krebs buffer (120 mM NaCl, 5 mM KCl, 2.5 mM CaCl_2_, 0.6 mM MgCl_2_, 0.6 mM KH_2_PO_4_, and 20 mM HEPES; pH 7.4) containing 2.2 mM glucose for 60 min at 37 °C. After pre-incubation, cells were incubated with the same buffer containing 20 mM glucose for 5 min and then with the same buffer containing 2.2 mM glucose for 5 min. To examine the involvement of microtubules in trafficking of insulin granules, cells were treated with 2 μM colchicine (FUJIFILM Wako Pure Chemical, 039-03851) for 15 min after pre-incubation. The cells were then rinsed with phosphate-buffered saline, fixed with 4% paraformaldehyde for 30 min, and permeabilized in phosphate-buffered saline containing 0.4% saponin, 1% bovine serum albumin, and 2% normal goat serum at room temperature for 15 min. The samples were incubated with primary antibodies against V5 (1:500), insulin (1:750), and α-tubulin (1:2000) at 4 °C overnight and then with fluorescent dye-conjugated secondary antibodies (1:200, Life Technologies) at room temperature for 1 h. Cells were then rinsed with phosphate-buffered saline three times and ultrapure water once before being mounted on microscope slide glasses using PermaFluor aqueous mounting medium (Richard Allan Scientific, TA-030-FM). Fluorescence images were acquired using an FV-1000 confocal microscope equipped with a 100 × objective, NA 1.40, and analyzed with FV10-ASW software (OLYMPUS). Using the acquired images, the cell peripheral area was defined as the region between the cell edge and 0.75 µm inside from this edge. The average signal intensity of insulin or V5-Rab27A in the cell peripheral area and the other area was calculated using ImageJ software. Cells in which the average signal intensity of insulin or V5-Rab27A in the cell peripheral area was more than twice that in the other area were defined as those with peripheral insulin or V5-Rab27A.

### Measurement of insulin secretion

MIN6 cells were plated at a density of 1 × 10^5^ cells/well in 48-well plates (Corning, 3548), cultured in DMEM containing 10% fetal bovine serum and antibiotics for 72 h, placed in HEPES-balanced Krebs Ringer bicarbonate buffer (KRH; 119 mM NaCl, 4.7 mM KCl, 2.5 mM CaCl_2_, 1.2 mM MgCl_2_, 1.2 mM KH_2_PO_4_, 25 mM NaHCO_3_, and 20 mM HEPES; pH 7.4) supplemented with 5 mg/mL bovine serum albumin for 2 h at 37 °C, and further incubated in fresh KRH containing various concentrations of glucose for 2 h at 37 °C. Thereafter, the medium was recovered and the cultured cells were harvested with phosphate-buffered saline. The cells were centrifuged at 900 × g for 5 min at room temperature and sonicated with phosphate-buffered saline. The total protein concentration of the sonicated cells was quantified using Micro-BCA Protein Assay Reagent (PIERCE Biotechnology, 23235). Cellular insulin was extracted with acid–ethanol for 20–24 h at 4 °C. The insulin content in the recovered medium and acid–ethanol extracts was determined using an insulin ELISA kit (Morinaga BioScience, M1108) according to the manufacturer’s protocol.

### Electron microscopy

MIN6 cells were plated at a density 2.5 × 10^6^ cells in a 60 mm dish and cultured in DMEM containing 15% fetal bovine serum and antibiotics for 72 h. The cells were placed in HEPES-buffered Krebs buffer containing 2.2 mM glucose for 2 h at 37 °C, rinsed twice with 0.1 M phosphate buffer pH 7.4, and fixed with buffer containing 2.5% glutaraldehyde, 2.0% paraformaldehyde, and 0.1 M phosphate overnight at 4 °C. The fixed samples were rinsed twice with 0.1 M phosphate buffer for 10 min. After osmification with buffer containing 1% osmium tetroxide and 0.1 M phosphate for 2 h at 4 °C, the samples were furthered dehydrated with a series of 50%, 70%, 80%, 90%, and 100% ethanol for 15 min each and embedded in Epon 812 (TAAB Laboratories, 3402). Ultrathin Sects. (70 nm) were cut using an ultramicrotome (Leica, EM-UC6) and stained with 1% uranyl acetate and lead citrate for 30 and 5 min, respectively. Images were captured at 100 kV with a transmission electron microscope (Hitachi, H-7650).

### Real‐time polymerase chain reaction

Total RNA was extracted from 5 × 10^6^ MIN6 cells using an RNeasy Micro Kit. The purity and concentration of RNA samples were assessed by performing spectrophotometric measurements at 260 and 280 nm. Reverse transcription was carried out in a 10 μL reaction mixture, which included total RNA (2 μg), 5 × PrimeScript Master Mix (2 μL; Takara, RR036A), and RNase-free water. Real‐time PCR were carried out in quadruplicate for every cDNA sample on a LightCycler system (Roche Diagnostics, 05815916001) using 2 μL of cDNA templates and SsoFast EvaGreen Supermix (Bio‐Rad, 1725200). Relative mRNA expression was determined using the ΔΔCt method with β-actin and glyceraldehyde-3-phosphate dehydrogenase (GAPDH) as normalization controls. The sequences of the primers used are listed in Supplementary Table 1.

### Quantification and statistical analysis

The specific signals in western blotting and immunostaining were subjected to densitometric analysis using ImageJ software. The F-test, the unpaired two-tailed Student’s t-test, and a two-way ANOVA followed by the Holm-Šídák multiple comparison test were performed using SigmaPlot software, version 15.0. *p* < 0.05 was considered statistically significant. All experiments were independently performed at least three times.

## Supplementary Information

Supplementary Information.

## Data Availability

The datasets used and/or analyzed during the current study are available from the corresponding author upon reasonable request.
