# Supplementary material for: The a subunit isoforms of V-ATPase are involved in glucose-dependent trafficking of insulin granules
Source: Sci Rep. 2025 May 30;15:18982. doi: 10.1038/s41598-025-02997-7 (PMC12125285; doi:10.1038/s41598-025-02997-7)
Supplement: Supplementary file 1 — Supplementary Information. [file 41598_2025_2997_MOESM1_ESM.pdf]

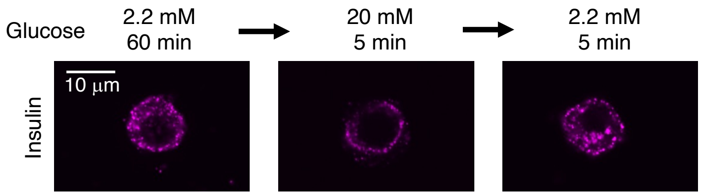

Supplementary Fig. 1

Supplementary Fig. 1. Localization of insulin granules in WT MIN6 cells. WT cells not expressing V5-Rab27A were sequentially incubated with 2.2 mM glucose for 60 min, 20 mM glucose for 5 min, and 2.2 mM glucose for 5 min, and then stained with an antibody specific for insulin (magenta).

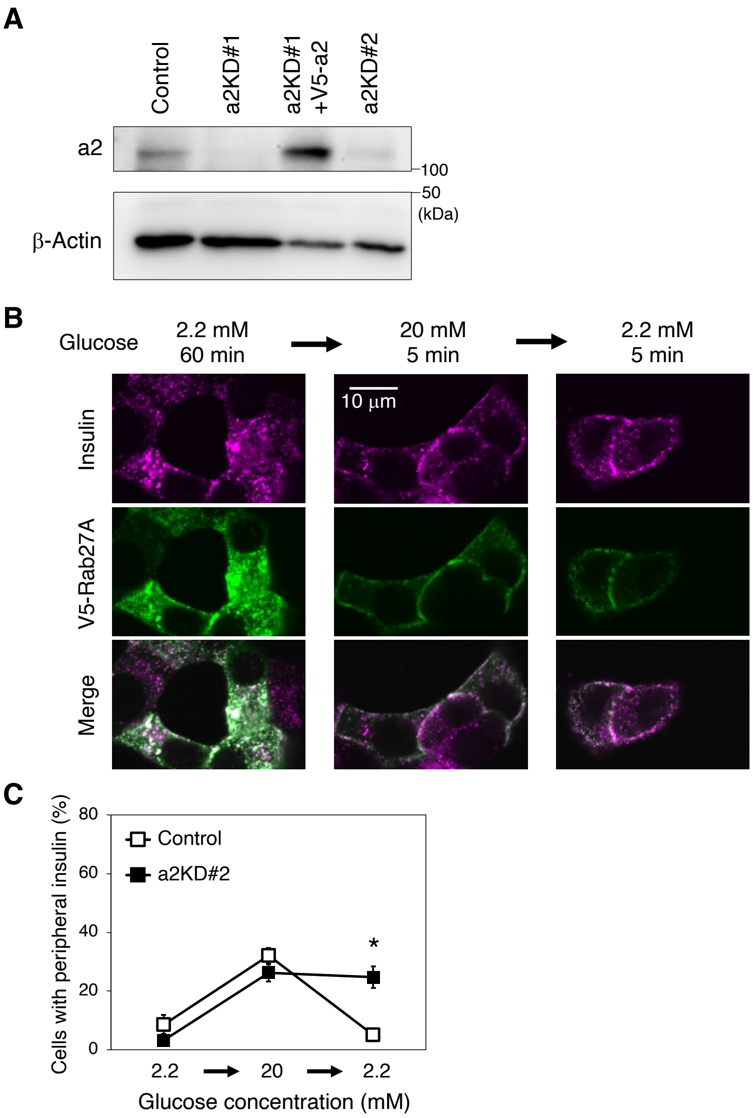

Supplementary Fig. 2

Supplementary Fig. 2. Localization of insulin granules in other a2KD MIN6 cells. **A** Expression of a2 in a2KD#2 cells. Cells were transduced with pSIREN-RetroQ-negative control shRNA (control) or pSIREN-RetroQ-a2-shRNA#2 (a2KD#2). a2 was detected with an antibody specific for a2 (upper panel). β-actin was also detected using a corresponding antibody (lower panel). Original blots are presented in Supplementary Fig. 10. The blots were cut horizontally at ~75 kDa. Upper and lower membranes were subjected to hybridization with anti-a2 and anti-β-actin antibodies, respectively. A full-length membrane was cut to conserve the anti-a2 antibody, which is commercially unavailable. **B** Localizations of insulin and V5-Rab27A in a2KD#2 cells. Cells expressing V5-Rab27A were sequentially incubated with 2.2 mM glucose for 60 min, 20 mM glucose for 5 min, and 2.2 mM glucose for 5 min, and then stained with antibodies specific for insulin (magenta) and V5 (green). Merged images are also shown. **C** Cells with peripheral insulin according to the glucose concentration. The percentage of cells with peripheral insulin was calculated as described in Fig. 2B. Control cells, open squares; a2KD#2 cells, closed squares. Data are means ± S.E. from three independent experiments. n > 30 cells in each experiment. *$p < 0.05$, unpaired multiple t test (Holm-Šídák method).

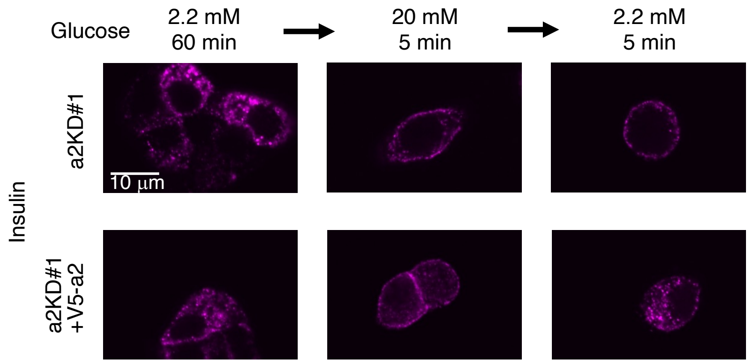

Supplementary Fig. 3

Supplementary Fig. 3. Localization of insulin granules in a2KD#1 and a2KD+V5-a2 MIN6 cells. Cells were sequentially incubated with 2.2 mM glucose for 60 min, 20 mM glucose for 5 min, and 2.2 mM glucose for 5 min, and then stained with an antibody specific for insulin (magenta).

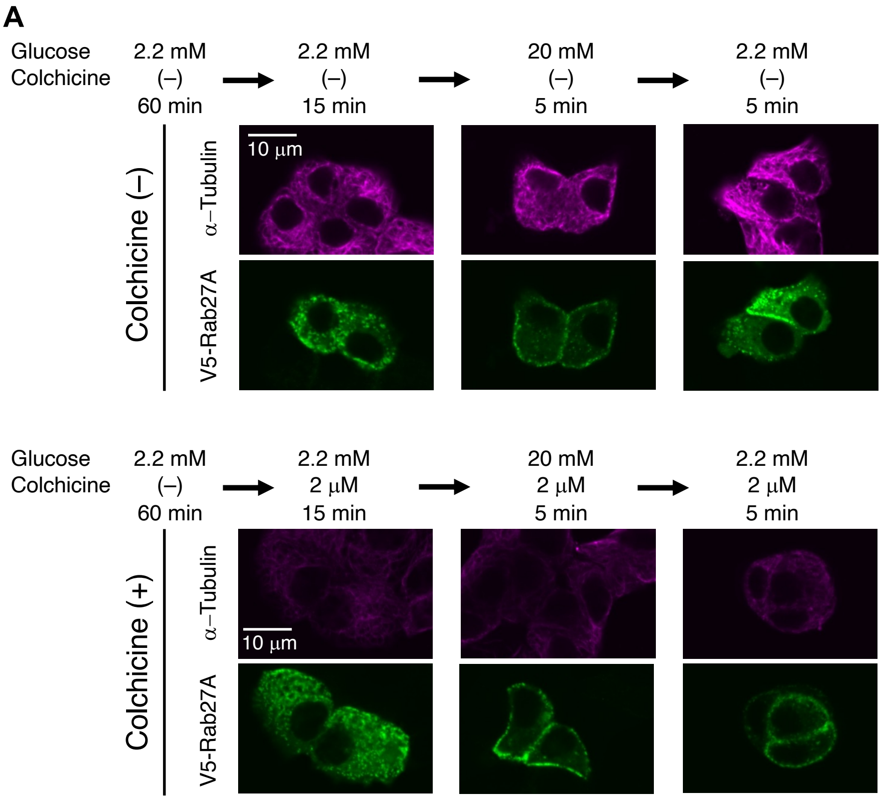

Supplementary Fig. 4

Supplementary Fig. 4. Localizations of microtubules and V5-Rab27A in MIN6 cells treated with colchicine. Cells expressing V5-Rab27A were preincubated with 2.2 mM glucose for 60 min, and then, in the absence (colchicine (–), upper panels) or presence (colchicine (+), lower panels) of 2 μM colchicine, sequentially incubated with 2.2 mM glucose for 15 min, 20 mM glucose for 5 min, and 2.2 mM glucose for 5 min. The treated cells were stained with antibodies specific for α-tubulin (magenta) and V5 (green).

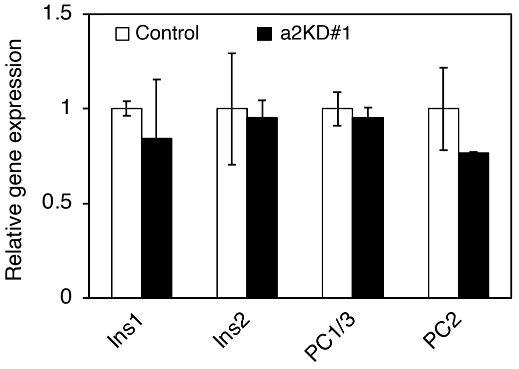

Supplementary Fig. 5

Supplementary Fig. 5 Expression levels of insulin and insulin-related genes in control and a2KD MIN6 cells quantified by real-time RT-PCR using glyceraldehyde-3-phosphate dehydrogenase (GAPDH) gene as an internal standard. Real-time RT-PCR was performed as described in Fig. 6B using GAPDH as an internal standard. Data are means ± S.E. from three independent experiments. There is no significant difference between the control and a2KD#1 cells by the unpaired two-tailed Student's t-test.

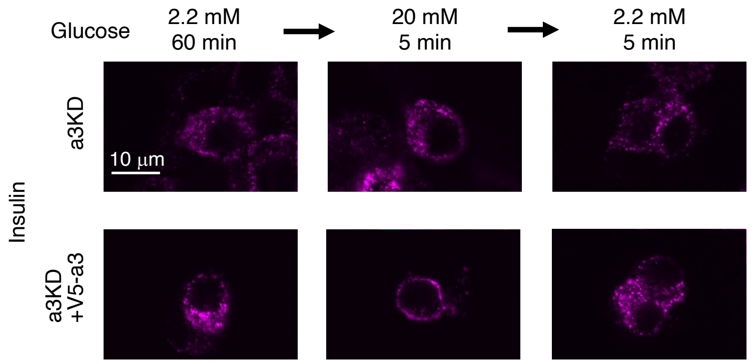

Supplementary Fig. 6

Supplementary Fig. 6 Localization of insulin granules in a3KD and a3KD+V5-a3 MIN6 cells. Cells were sequentially incubated with 2.2 mM glucose for 60 min, 20 mM glucose for 5 min, and 2.2 mM glucose for 5 min, and then stained with an antibody specific for insulin (magenta).

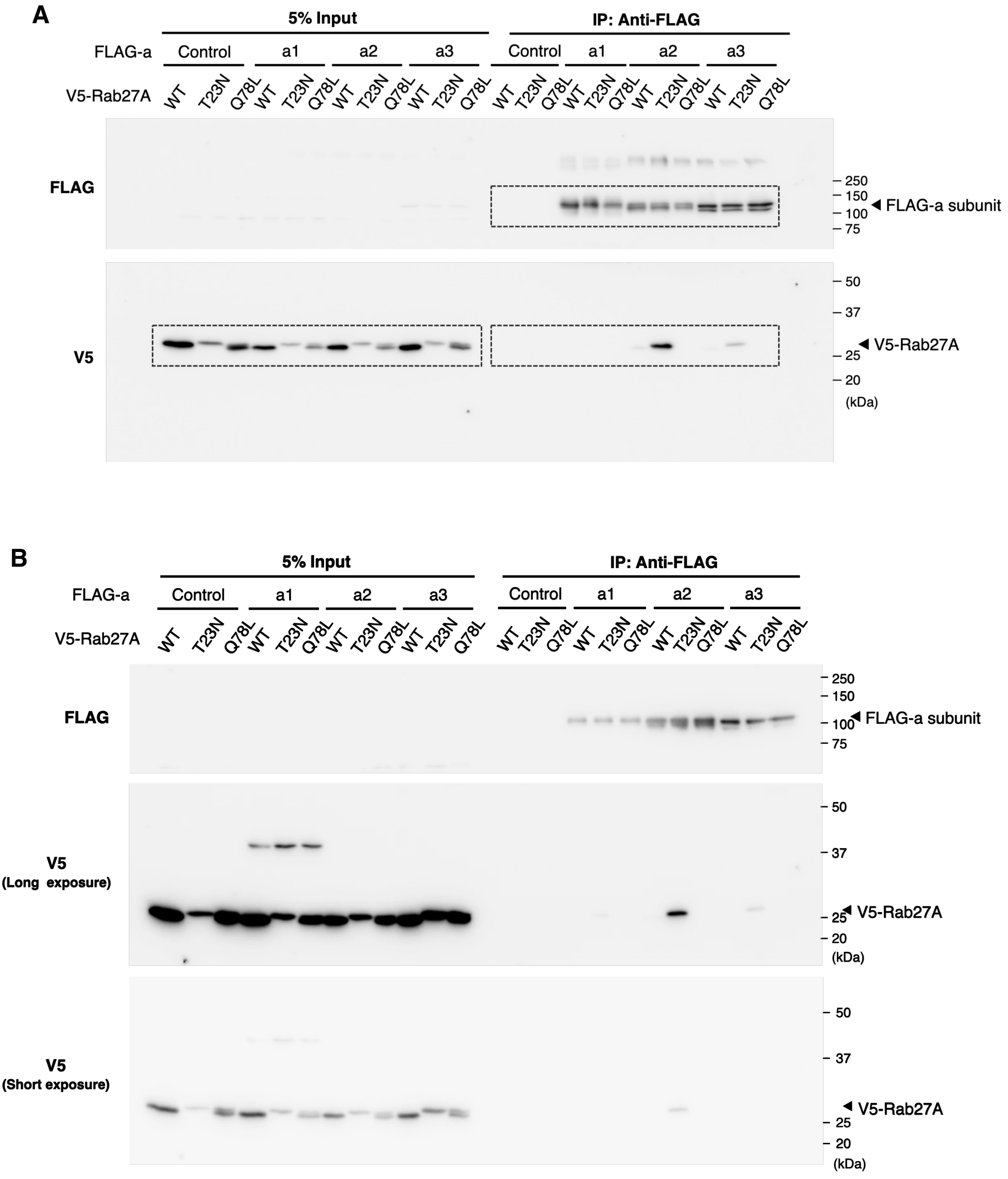
A
5% Input
IP: Anti-FLAG
FLAG-a
Control
a1
a2
a3
V5-Rab27A
WT
T23N
Q78L
FLAG
V5
250
150
100
75
50
37
25
20
(kDa)
FLAG-a subunit
V5-Rab27A
B
5% Input
IP: Anti-FLAG
FLAG-a
Control
a1
a2
a3
V5-Rab27A
WT
T23N
Q78L
FLAG
V5
(Long exposure)
V5
(Short exposure)
250
150
100
75
50
37
25
20
(kDa)
FLAG-a subunit
V5-Rab27A

C

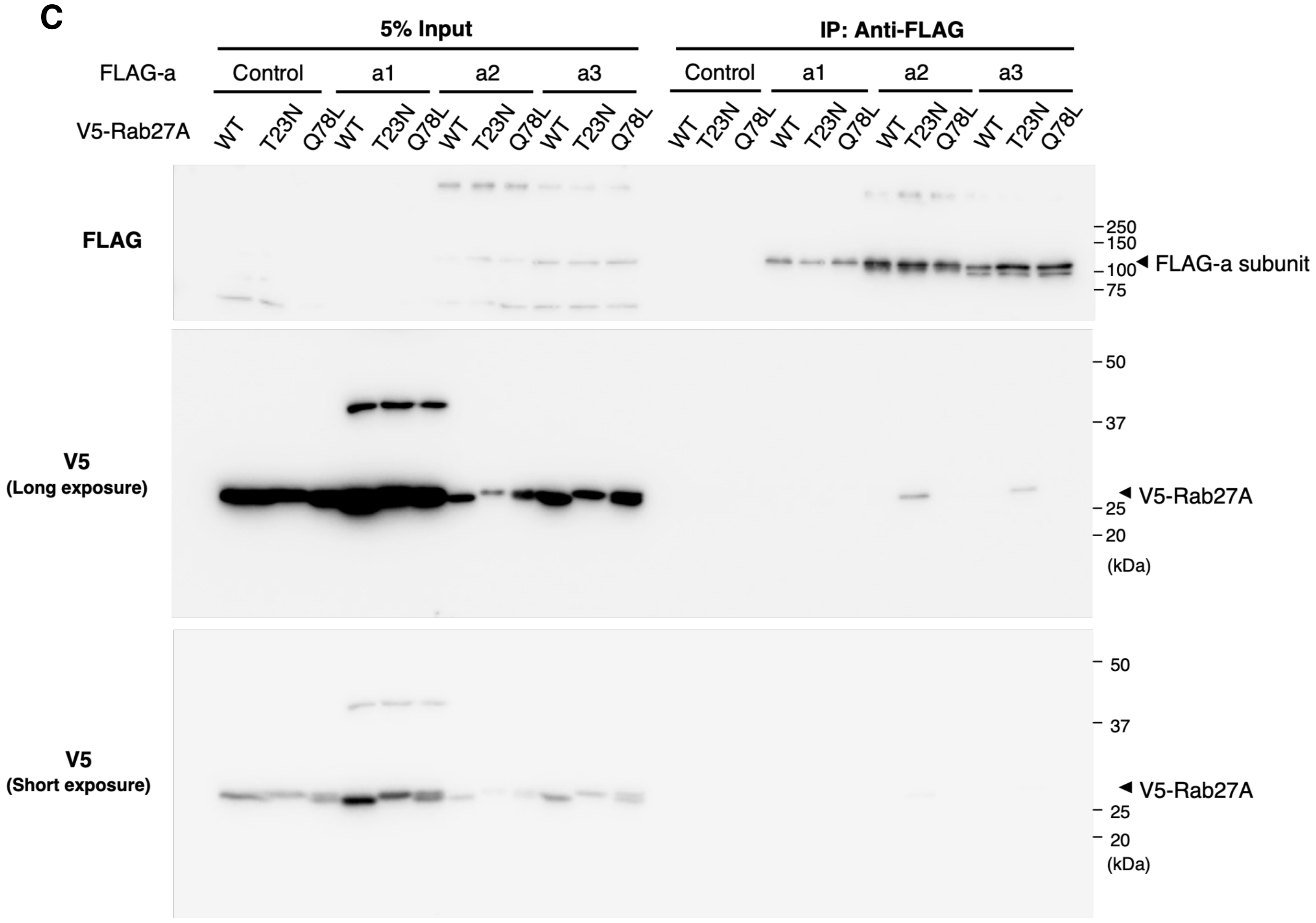

Supplementary Fig. 7

Supplementary Fig. 7 A Original blots of Fig. 1B. The blots were cut horizontally at ~60 kDa. Upper and lower membranes were subjected to hybridization with anti-FLAG and anti-V5 antibodies, respectively. A full-length membrane was cut because the amount of protein in immunoprecipitation samples was insufficient to prepare multiple full-length membranes. **B–C** Images of blots for all replicates.

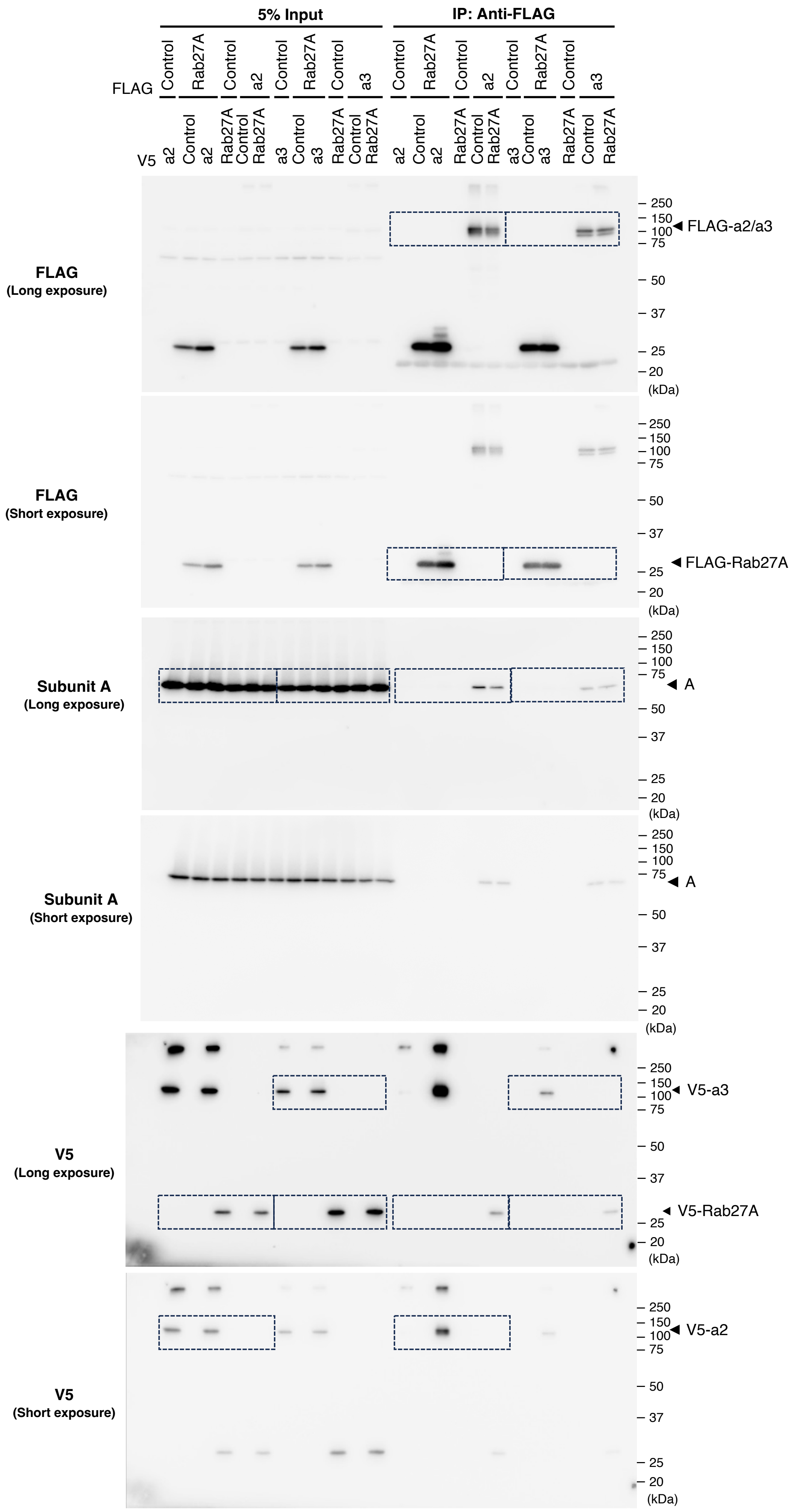

Supplementary Fig. 8

Supplementary Fig. 8. Original blots of Fig. 1C, D.

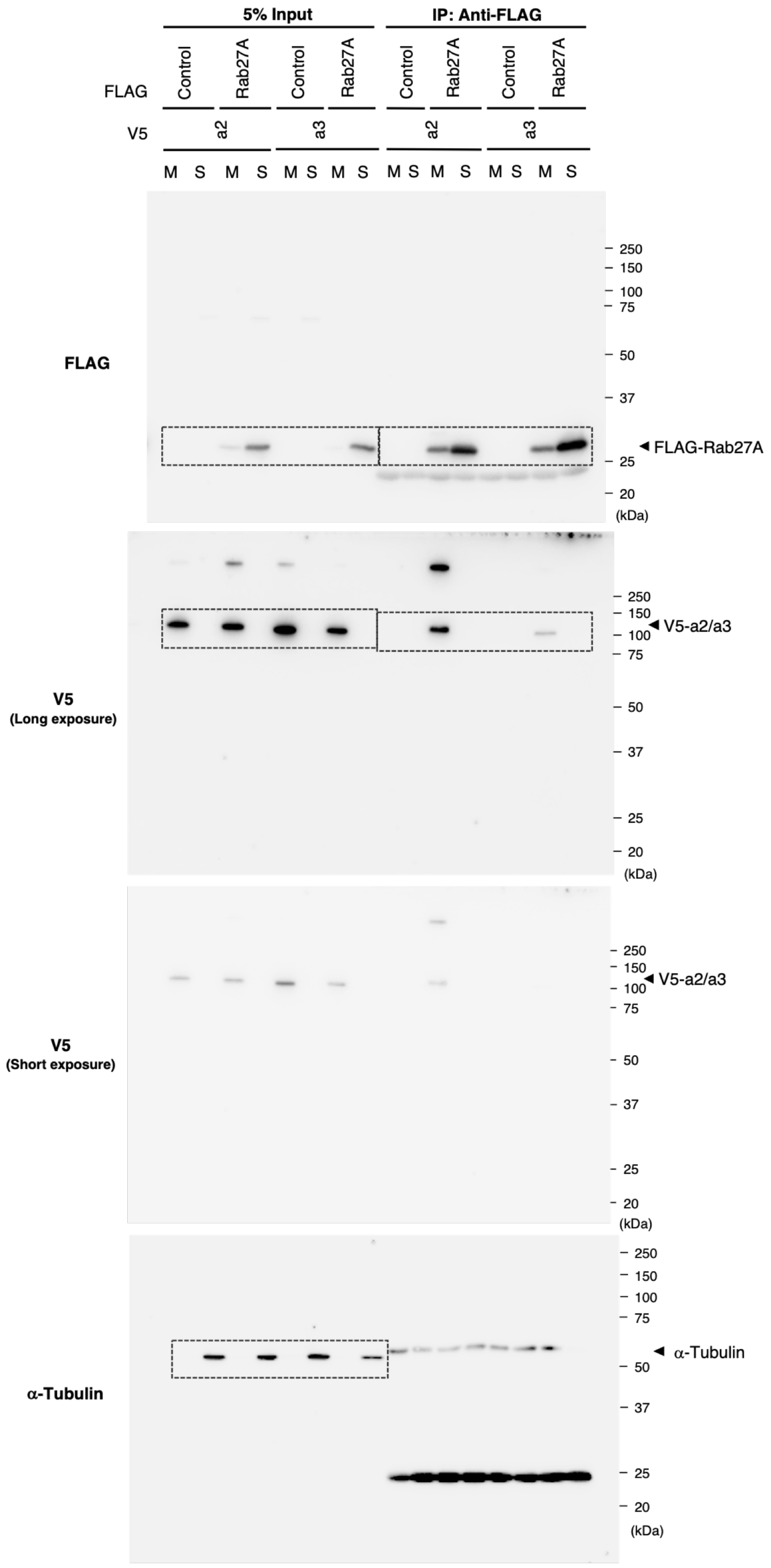

Supplementary Fig. 9

Supplementary Fig. 9. Original blots of Fig. 1E.

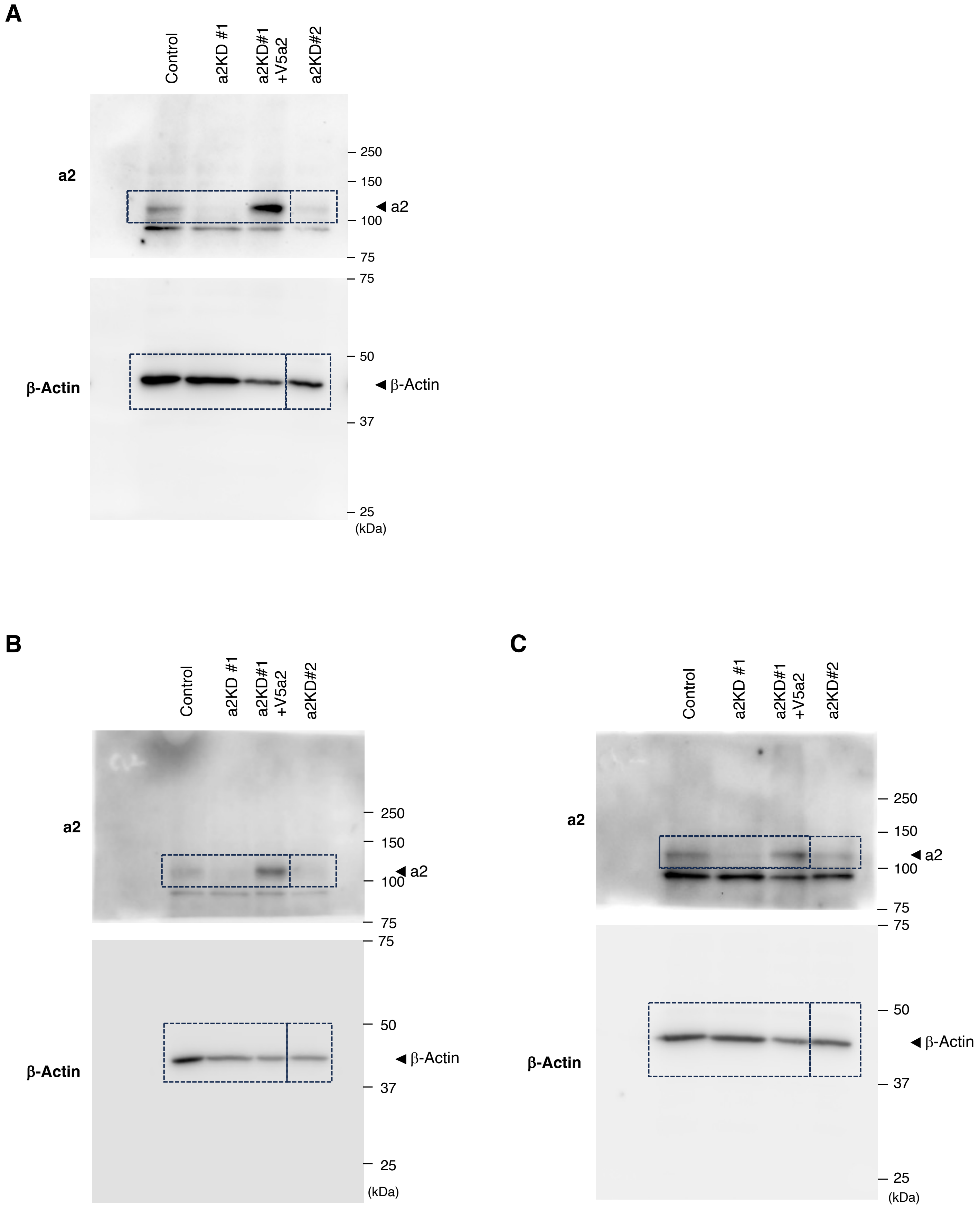

Supplementary Fig. 10

Supplementary Fig. 10. **A** Original blots of Fig. 3A and Supplementary Fig. 2A. The blots were cut horizontally at ~75 kDa. Upper and lower membranes were subjected to hybridization with anti-a2 and anti-β-actin antibodies, respectively. A full-length membrane was cut to conserve the anti-a2 antibody, which is commercially unavailable. **B**,**C** Images of blots for all replicates.

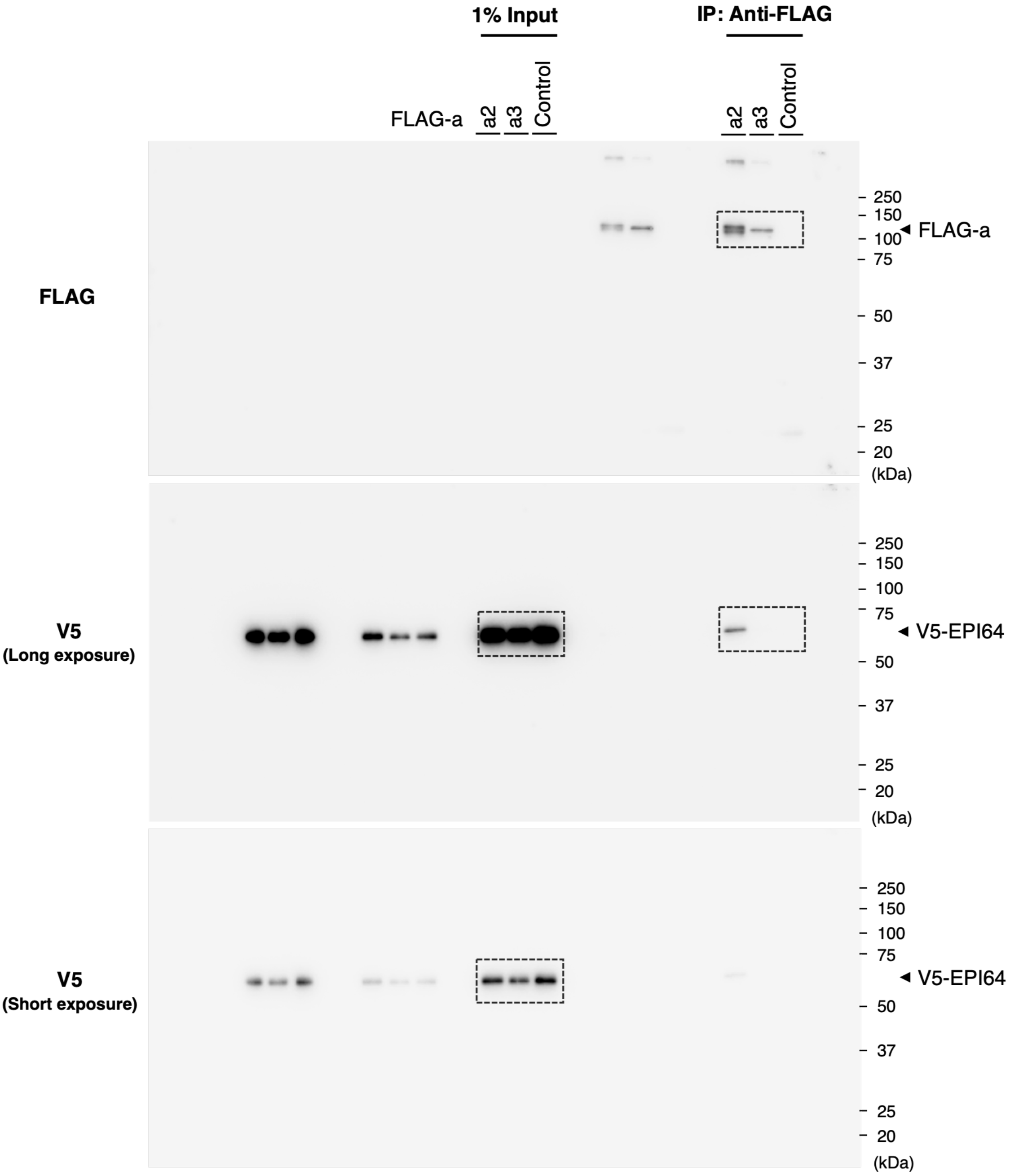

Supplementary Fig. 11

Supplementary Fig. 11. Original blots of Fig. 6.

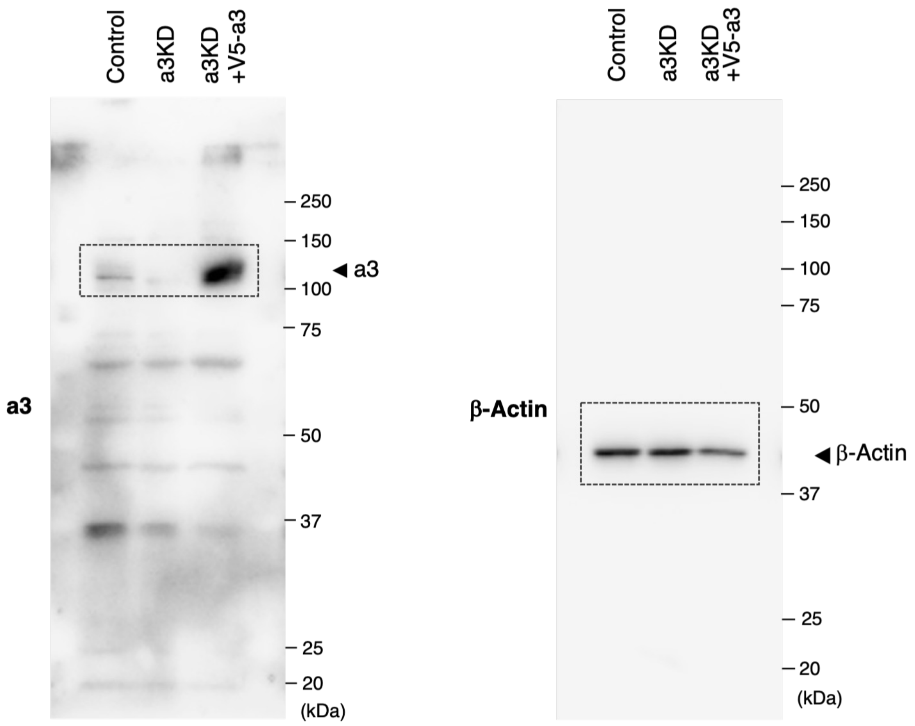

Supplementary Fig. 12

Supplementary Fig. 12. Original blots of Fig. 7A.

Supplementary Table 1. Sequences of oligonucleotides used in this study

Oligonucleotide	5’→3’ sequence
EPI64 FW	GAATTCGGATCCGCGAAAAGCAGCAGAGAG
EPI64 RV	GAATTCGCGGCCGCCAAGTAGGTGTCCTCACT
shRNA a2KD#1 S	GATCCGGCTCAATACTCGGATCCAATTCAAGAGATTGGATCC GAGTATTGAGCCTTTTTTGCGGCCGCG
shRNA a2KD#1 A	AATTCGCGGCCGCAAAAAAGGCTCAATACTCGGATCCAATC TCTTGAATTGGATCCGAGTATTGAGCCG
shRNA a2KD#2 S	GATCCGGCAGGAGTGTAGTAAGGCATTCAAGAGATGCCTTA CTACACTCCTGCCTTTTTTGCGGCCGCG
shRNA a2KD#2 A	AATTCGCGGCCGCAAAAAAGGCAGGAGTGTAGTAAGGCATC TCTTGAATGCCTTACTACACTCCTGCCG
shRNA a3KD S	GATCCGCGGACTGCTCATGTTTCTCTTTTCAAGAGAAAGAGA AACATGAGCAGTCCGCTTTTTTGCGGCCGCG
shRNA a3KD A	AATTCGCGGCCGCAAAAAAGCGGACTGCTCATGTTTCTCTTT CTCTTGAAAAGAGAAACATGAGCAGTCCGCG
Ins1 FW	TAGTGACCAGCTATAATCAGAG
Ins1 RV	ACGCCAAGGTCTGAAGGTCC
Ins2 FW	CCCTGCTGGCCCTGCTCTT
Ins2 RV	AGGTCTGAAGGTCACCTGCT
PC1/3 FW	ATGGAGCAAAGAGGTTGGAC
PC1/3 RV	GCTGCAGTCATTCTGGTATC
PC2 FW	TCGCCAAGTTGCAGCAGAAC
PC2 RV	CTTCGGCCACGTTCAAGTCTA
β-Actin FW	ACAGCTTCTTTGCAGCTCCTTC
β-Actin RV	CCCATTCCCACCATCACAC
GAPDH FW	TCTGGAAAGCTGTGGCGTGATG
GAPDH RV	GGTGCACGAACTTTATTGATGG
